# Homogeneous catalysis in continuous flow integrating photocatalysis, electrocatalysis, and automation technologies

**DOI:** 10.1038/s42004-025-01725-6

**Published:** 2025-11-07

**Authors:** Laura F. Peña, Lucía G. Parte, Carlos Díez-Poza, Javier Guerra, Enol López

**Affiliations:** 1 Faculty of Pharmacy, University of Castilla-La Mancha, Albacete, Spain; 2 Department of Organic Chemistry, Science Faculty, University of Valladolid (UVa), Valladolid, Spain; 3 Department of Organic Chemistry, ITAP, School of Engineering (EII), University of Valladolid (UVa), Valladolid, Spain

**Keywords:** Catalysis, Flow chemistry, Synthetic chemistry methodology

## Abstract

In recent years, homogeneous catalysis in continuous flow has undergone remarkable advances, driving significant progress across a broad range of chemical transformations. In this review, we examine how novel synthetic tools such as photo- and electrocatalysis have been merged with catalytic processes to unlock synthetic opportunities and enable transformations in flow that are challenging under conventional batch conditions. Furthermore, we discuss the integration of automation and high-throughput methodologies, emphasizing their roles in synthesis, catalyst screening and reaction optimization in homogeneous flow catalysis. By providing a unified perspective on these developments, we highlight the impact of modern technologies and the potential for interdisciplinary innovation.

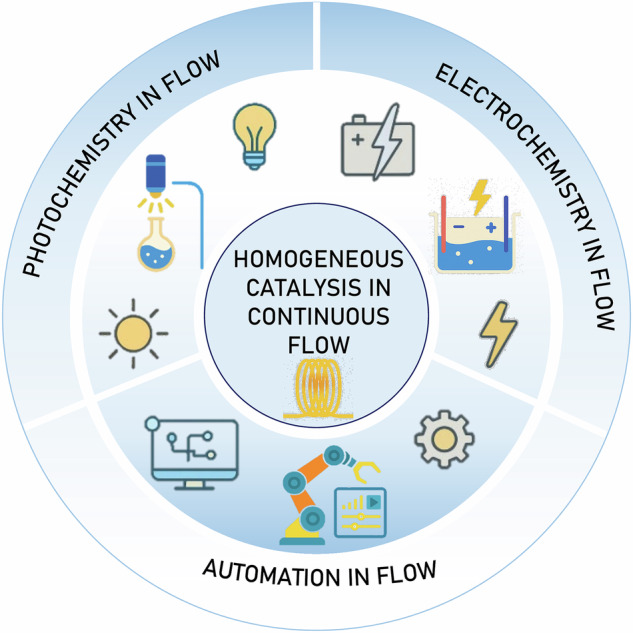

## Abstract (web summary)

Recent advances in homogeneous catalysis in continuous flow have revolutionized chemical transformations. Here, the authors review the integration of continuous flow with photo- and electrocatalysis, automation and high-throughput methods, highlighting their potential to overcome synthetic challenges in batch processes.

## Introduction

Catalytic methodologies provide new opportunities to access underexplored chemical spaces that are sometimes inaccessible because of the high activation barriers of chemical reactions. Depending on the catalyst employed, both homogeneous and heterogeneous systems have been developed^1,2^. Heterogeneous catalysts are usually stable and can be recycled, but sometimes lack activity or selectivity^3^, while homogeneous catalysts have demonstrated very high activities for specific transformations, by improving yields and shortening reaction times^4^.

In recent years, both approaches have been implemented in continuous flow chemistry, an innovative tool in organic synthesis that represents a paradigm shift aimed at achieving the objectives described by the Principles of Sustainable Chemistry^5–7^. This continuous manufacturing approach aims to carry out the synthesis of chemical substances in an environmentally friendly manner, starting from their design and development^8–12^, and enables implementation of systematic methodologies in the pharmaceutical sector, known as "Quality by Design (QbD)"^13^. This is particularly relevant for the synthesis of drugs and Active Pharmaceutical Ingredients (APIs), where stringent quality control is essential for patient safety and regulatory compliance.

The intrinsic advantages of flow chemistry are maintained across all production scales, enabling precise control of critical parameters such as temperature, pressure, mixing, and residence time^14,15^. Mass and energy transfers are higher than in traditional batch processes^9^, contributing to improved selectivity, yields, and product quality. Moreover, flow chemistry is designed to safely operate under extreme reaction conditions, such as elevated pressures and temperatures, facilitating Process Intensification- achieving the same or superior purity, selectivity, and yield in shorter reaction times at larger scales, thereby enhancing productivity and reducing environmental impact^16,17^.

To ensure precise control and process consistency, the integration of Process Analytical Technology (PAT) tools is essential in continuous processing^18–21^. These enable real-time monitoring and control of both critical parameters and product quality^18,22–24^. Inline monitoring, where analytical instruments are integrated directly into the process stream, provides continuous, non-destructive data without manual sampling. Online monitoring, by contrast, involves automated periodic sampling via a bypass line for off-line analysis, offering complementary insights for optimization^25^. The adoption of continuous manufacturing supported by these tools has led to the development of specific regulatory guidelines for the pharmaceutical sector^26^.

Flow chemistry offers more predictable and efficient scale-up than batch, leveraging its high surface-to-volume ratio for superior heat and mass transfer^14,27–29^. Scalability is achieved through prolonged operation, "numbering-up" (multiplying units), "sizing-up" (increasing reactor volume via channel modifications)^14,28^ or "smart dimensioning," a hybrid strategy preserving micro-environment benefits while adapting reactor geometry for larger throughputs. Minimized reactor volumes and the in situ generation of unstable reagents enhance safety^8,30^, whereas the integration of real-time monitoring and automation ensures scalability with minimal re-optimization.

Beyond conventional transformations, flow chemistry accommodates techniques rarely implemented industrially in batch, such as photochemistry^31,32^, electrochemistry, sonochemistry^33^ or microwave-assisted chemistry^34–36^. This versatility expands the synthetic toolbox to unlock novel reactivities under well-controlled and scalable conditions.

Among the various innovations contributing to the sustainability of continuous flow catalysis, the adoption of green and cost-effective reagents stands out, in which photo- and electrochemistry have become essential tools^7,37^. Photochemistry contributes to the development of sustainable chemical processes using light as energy source to construct new chemical entities^38^, whereas electrosynthesis makes use of electrical energy to generate active intermediates^39–44^. Photoredox catalysis harnesses photon energy, while electrosynthesis utilizes the potential energy between electrodes to drive the formation of radicals or ions. A key distinction between these methods lies in the spatial separation of oxidation and reduction sites. In photoredox catalysis, a freely diffusing photocatalyst mediates both oxidation and reduction within the solution. In contrast, electrosynthesis confines these reactions to the electrode surfaces of an electrochemical cell, offering a structured and controllable environment for radical generation. Despite the great number of advantages of using both approaches, limitations of the mass or energy transfer processes have limited broader use. An appropriate alternative is the combination of photochemistry and/or electrochemistry with continuous flow technology, highly convenient to overcome these limitations in batch or to scale-up photochemical^45–49^ or electrochemical protocols^50–52^.

In parallel with these advances in catalytic methodology, chemistry has benefitted from technological progress with the development of new flow reactors, photocatalytic systems and electrochemical cells^53^. In addition, computer science has enabled the development of automated chemical processes such as reaction planning, optimization, synthesis and analysis through integrated inline analytical techniques^25,54,55^.

**Fig. 1:**
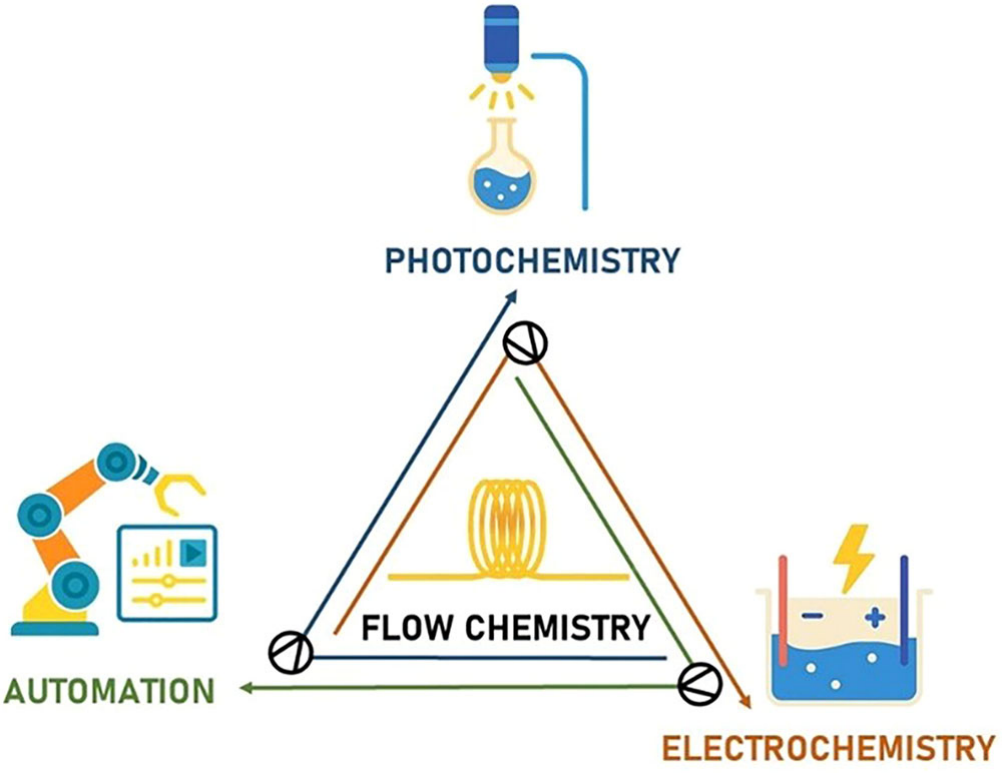
Overview of modern strategies in flow chemistry: photochemistry, electrochemistry, and automation. The combination of technologies like flow chemistry, photocatalytic systems, electrochemical cells, and automation shapes the field of modern homogeneous catalysis.

In this review, we discuss how the particular field of homogeneous catalysis in continuous flow has been merged with photocatalysis, electrocatalysis, and automation strategies to offer several advantages in the construction of valuable organic scaffolds through more efficient methodologies (Fig. 1). The following sections examine the main advances in each field, highlighting key developments within continuous flow systems.

## Main text

### Photocatalysis in continuous flow

Photochemistry plays a significant role in advancing sustainable chemical processes^56^. Redox-modulating techniques enable chemists to selectively target functional groups within a molecule based on their distinct redox potentials, facilitating the mild and selective generation of radicals. Unlike conventional two-electron chemistry, radical-based approaches introduce alternative reactivity and selectivity profiles, often simplifying synthesis through non-traditional bond construction. This renewed interest in radicals has driven their integration into modern chemical synthesis.

Reactions involving radical intermediates can be classified as net oxidations, net reductions, or redox-neutral processes. In the first two cases, stoichiometric reagents act as electron donors or acceptors. However, in redox-neutral reactions, the balance of electron-transfer steps between molecular partners enables the efficient construction of complex molecules with improved atom economy. One such approach involves transforming both reactive partners into free-radical intermediates, allowing bond formation through radical-radical coupling. The primary activation barrier in these reactions arises from oxidation and reduction steps, which can be overcome using photoredox catalysis or electrosynthesis.

Light (photons) can be considered a reactant in chemical reactions, making it one of the cleanest and most renewable energy sources available for the synthesis of new chemical entities. However, the application of photochemistry in synthetic organic chemistry has remained limited until the past two decades and is still relatively unfamiliar to many. Several factors contribute to this state of affairs: (1) limited knowledge regarding the required equipment, (2) safety concerns related to the high operating temperatures of mercury (Hg) lamps and the potential harm from UV radiation, and (3) challenges in scaling up reactions using traditional immersion-well reactors. Even within the industry, large-scale photoreactions are rare, often deemed inefficient and slow, and frequently lead to product decomposition due to over-irradiation. The primary cause of this inefficiency lies in the logarithmic decrease in light transmission through a liquid medium with increasing path length, as described by the Lambert-Beer Law. The amount of photon flux required is scale-dependent, since irradiation occurs only within a limited radius around the light source. High concentrations of photochemically active species can further shrink the illuminated zone, reducing the uniformity of the solution's exposure to the photons emitted. Consequently, concentrated solutions in batch reactors can promote side reactions, and lower concentrations are often preferred to ensure more uniform irradiation^46,57^.

**Scheme 1:**
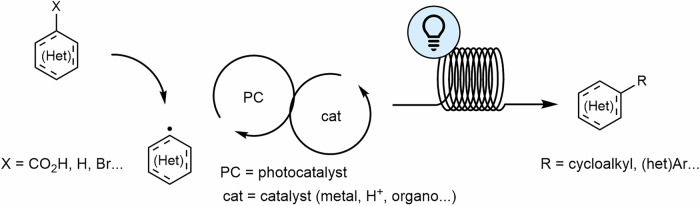
**A general scheme of flow photocatalyzed reactions**. In a first step, a photocatalyst activates the organic substrate. Then, further catalysis with transition metals (metallaphotoredox), organocatalysts, or Lewis acids, provides access to a wide range of products. PC photocatalyst, cat catalyst (metal, H^+^, organo…).

The advantages of radical-based photochemical reactions can be increased in continuous flow systems. Particularly in microreactors with narrow tubing, these transformations are significantly more efficient than those performed in batch processes. This efficiency stems from the shorter distance between the light source and the reaction medium, leading to more effective irradiation. The homogeneous photon flux in flow systems enables reproducible and faster chemical processes. Moreover, the continuous removal of products reduces degradation by preventing over-irradiation, which is common in batch processes. Additionally, the use of visible light in photoredox catalysis has broadened the range of achievable transformations. In photoredox catalysis, transition metal-based or organic photocatalysts absorb visible light to activate organic substrates via one-electron transfer (Scheme 1). These intermediates can participate in subsequent catalytic transformations promoted by transition metals (metallophotoredox), organocatalysis or Lewis Acids or they can be coupled with different reactive agents^14,46,47,57–68^. Photocatalysis offers several undeniable advantages, including the use of light as an inexpensive and harmless reagent, the ability to perform reactions under mild conditions, and a versatile approach (on/off) that helps minimize the formation of impurities.

#### C(sp²)–C(sp³) cross-electrophile coupling reaction

**Scheme 2:**
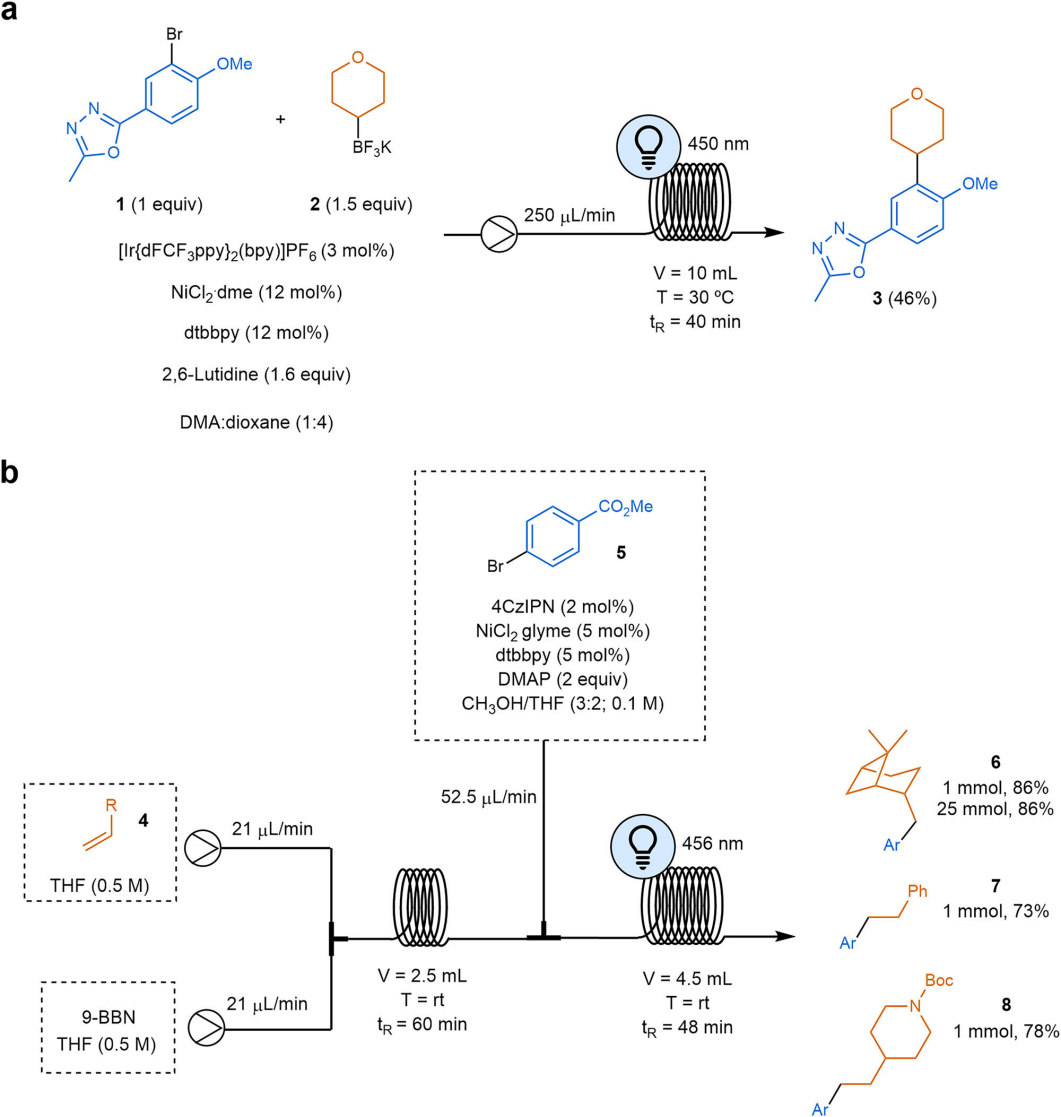
**Examples of photocatalyzed C(sp²)–C(sp³) cross-electrophile coupling reactions in flow. a** Coupling reaction of sterically hindered o-methoxy aryl bromide **1** with potassium trifluoroborate **2**. dtbbpy: 4,4'-di-tert-butyl-2,2'-bipyridine; t_R_: residence time. **b** C(sp^2^)–C(sp^3^) Suzuki–Miyaura cross-coupling reaction of aryl bromides with alkyl boranes. 4CzIPN: 2,4,5,6-tetra(9H-carbazol-9-yl)isophthalonitrile; 9-BBN: 9-borabicyclo[3.3.1]nonane; glyme: dimethoxyethane.

Recently, a range of C(sp²)–C(sp³) cross-coupling reactions have been reported that utilize a combination of visible light photoredox catalysts and transition metal catalysts, such as nickel^69^. This approach combines the photochemical activation of the reactants with transition metal catalysis to facilitate the formation of carbon-carbon bonds. Unlike traditional palladium-catalyzed reactions, which rely on β-hydride elimination and transmetalation between aromatic and aliphatic compounds, this method leverages the unique synergy between photochemistry and transition metal catalysis. In this cross-electrophile C(sp²)–C(sp³) and C(sp³) –C(sp³) couplings, the nickel catalyst plays an essential role in trapping radicals generated by the photocatalyst. This entrapment involves the oxidation of Ni(II) to Ni(III). Reductive elimination affords the cross-coupled product regenerating the nickel catalyst. A comprehensive comparison between traditional and metallaphotoredox processes has been conducted, revealing that no single methodology is universally applicable. Each methodology offers distinct advantages and limitations, depending on the reaction conditions and substrates involved^70^. As stated by Speckmeier and Maier^69^, their general applicability is challenged by the direct link between the oxidation potential of each individual substrate and the driving force of the reaction (as described by the Rehm–Weller equation)^71^. These authors use dual photoredox (iridium photocatalyst)/nickel catalysis assisted by amino radical transfer (ART) key step in a boron-alkyl Suzuki–Miyaura cross-coupling process, providing this methodology with air and water stability and a wide scope of examples. In this work, the source of the alkyl radicals had its origin in boronic pinacol esters through the interaction of an amino radical with the vacant *p*-orbital of a boronic ester, leading to a rapid homolytic cleavage of the C–B bond and release of an alkyl radical. Boronic esters have alternatively been used as substrates instead of more common trifluoroborate salts due to their greater solubility in low-boiling-point solvents. Nevertheless, the procedure was limited to the coupling of benzyl and allyl boronic esters. A very close methodology has also been reported recently^72^ in which authors were able to apply an organic photocatalyst instead of an iridium derivative and scale the process under flow conditions based on previous studies for similar transformations^73–75^. Prior work on this transformation using trifluoroborate salts^74^ instead of boronic pinacol esters hindered the application of flow chemistry, as clogging issues were immediately observed in the flow equipment due to the rapid precipitation of insoluble potassium salts^75^. Nevertheless, careful optimization of the base and solvent enables this coupling process using trifluoroborate salts catalyzed by iridium/nickel cocatalysts^76^. The coupling of sterically hindered *o*-methoxy aryl bromide (**1**) with potassium trifluoroborate (**2**) resulted in only trace conversion to the product under both traditional heterogeneous and homogeneous batch conditions. However, when the reaction was conducted in a continuous flow setup, approximately 90% consumption of the starting material was achieved within 40 min, yielding compound **3** in 46% isolated yield (Scheme 2a). The success of this procedure highlights the importance of continuous flow to achieve a uniform and increased light exposure to perform the transformation on challenging substrates, which cannot react otherwise^76^.

Broadening the scope of this Suzuki–Miyaura cross-coupling process, Noël and coworkers^77^ recently reported the use of alkyl boranes synthesized through the *anti*-Markonikov regioselective hydroboration, resulting in a straightforward methodology to functionalize alkyl chain termini. Optimization of both processes makes possible the development of a telescoped process starting from the alkene, the boron derivative and the components necessary to perform the subsequent C(sp^2^)–C(sp^3^) cross-electrophile coupling reaction (Scheme 2b). Primary and secondary alkyl boronic acids as radical precursors were also used as starting materials for this transformation by means of a halogen transfer mechanism where the bromine radical (formed in situ via a photo-induced homolysis of the Ni–Br bond) and the empty *p*-orbital on the boron atom played a crucial role to photogenerate the alkyl radical that will enter in the oxidative addition step in the nickel cycle^78^.

**Scheme 3:**
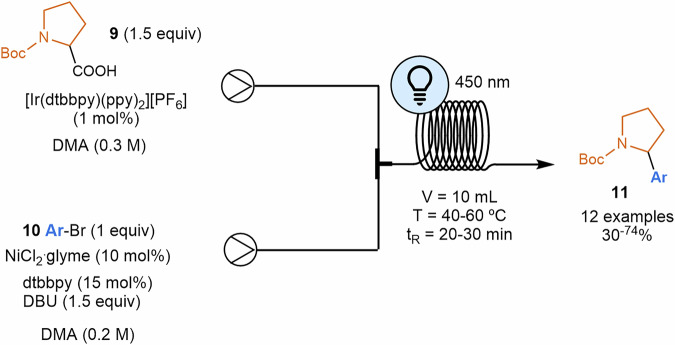
**Decarboxylative coupling reaction of amino acids with aryl halides in flow**. The space-time yield in flow was 430 times higher than under batch conditions.

In a different transformation, along with these lines, Abdiaj and Alcázar translated the methodology described by McMillan to perform the decarboxylative coupling of amino acids **9** with aryl halides **10**^73^ to a flow procedure (Scheme 3)^79^. After systematically optimizing the conditions for this transformation, with a focus on selecting the appropriate base and solvent compatible with flow instrumentation, the authors conducted a comparative study under optimal conditions, contrasting batch and flow processes. The results revealed that the space-time yield was 430 times higher under flow conditions. Special attention was again given to replacing the non-soluble, non-nucleophilic inorganic base with a soluble organic base. However, the resulting conjugate acid generated during the reaction often proved insoluble, making it difficult to avoid suspensions. This challenge was also encountered by Jensen, Robinson, and their colleagues, who utilized a segmented flow ("microslug") reactor with a specially designed photochemistry module for reaction screening and optimization. This system enabled to develop a self-optimizing algorithm, identifying optimal flow conditions for a model reaction by considering both continuous variables (temperature and time) and discrete variables (base and catalyst)^80^.

**Fig. 2:**
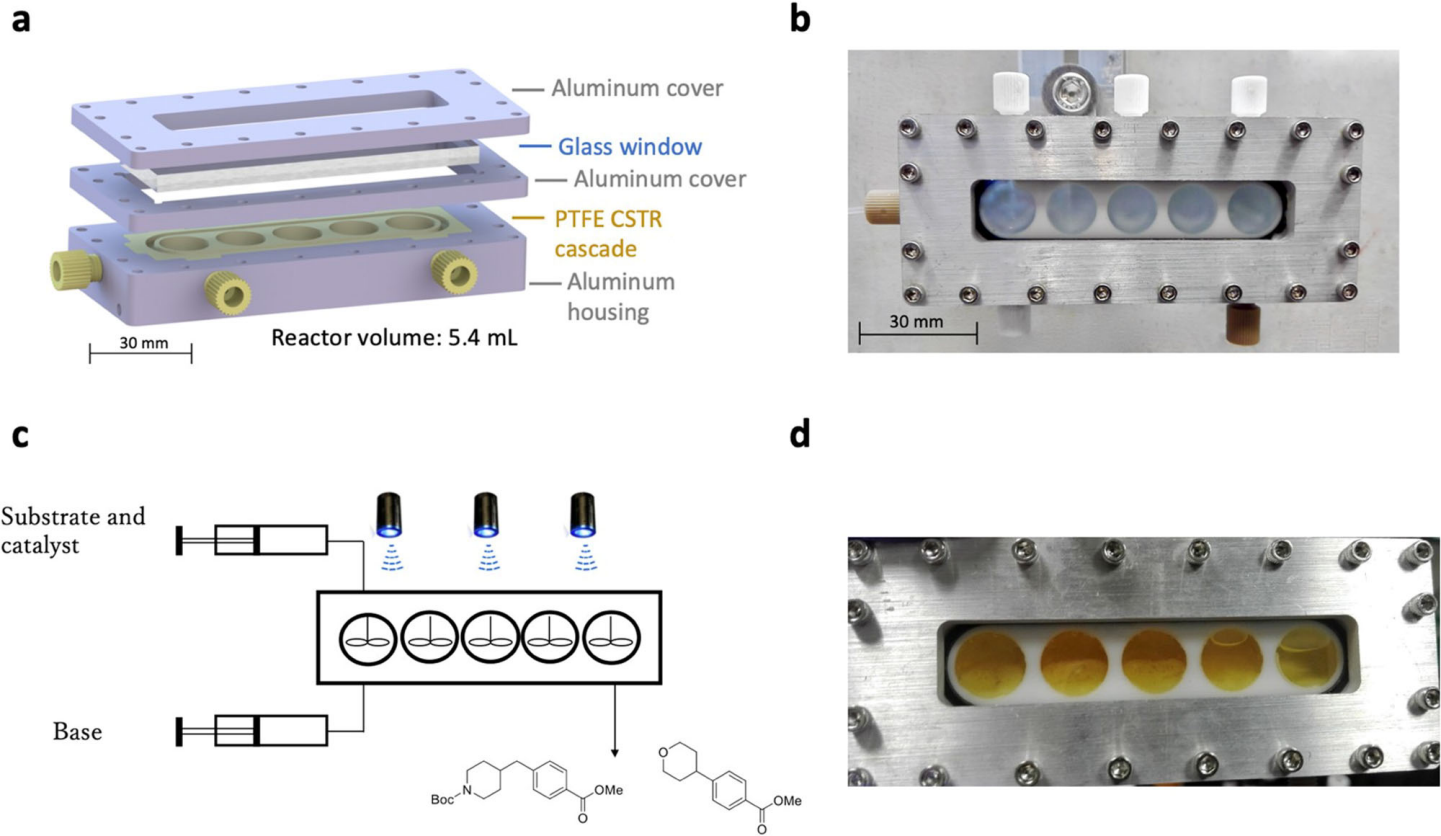
Microslug reactors used in segmented flow. **a** Exploded-view computer-aided design (CAD) model of the CSTR cascade for handling solid-containing photochemical reactions. **b** Picture of the assembled CSTR cascade. **c** Schematic of continuous flow synthesis in the CSTR. **d** CSTR after 8 h of continuous flow. It is visible that the solid byproduct makes up almost 50% in volume. "Adapted from Duvadie, R.; Pomberger, A.; Mo, Y.; Altinoglu, E. I.; Hsieh, H.-W.; Nandiwale, K. Y.; Schultz, V. L.; Jensen, K. F.; Robinson, R. I. Photoredox iridium-nickel dual catalyzed cross-electrophile coupling: from a batch to a continuous stirred-tank reactor via an automated segmented flow reactor. Org. Process Res. Dev., 25 (10), 2323–2330, Copyright (2021), with permission from American Chemical Society”.

A different approach has been employed by MacMillan and coworkers showing that the increase of the tubing dimension of a modified plug-flow reactor (PFR) setup is capable of handling the formation of these solids together with a new set of optimal conditions that were performed by means of a high-throughput experimentation platform^81^. In a continuous effort to increase the knowledge of the cross-electrophile coupling between aryl bromides and primary alkyl bromides, Jensen, Robinson and coworkers used their segmented flow (“microslug”) reactor to find the optimal conditions at 15 μL scale, and subsequently, transferred to a 5 mL photo-continuous stirred-tank reactor (CSTR) cascade to demonstrate a multigram continuous flow synthesis during a 24 h steady operation (Fig. 2)^82^.

**Scheme 4:**
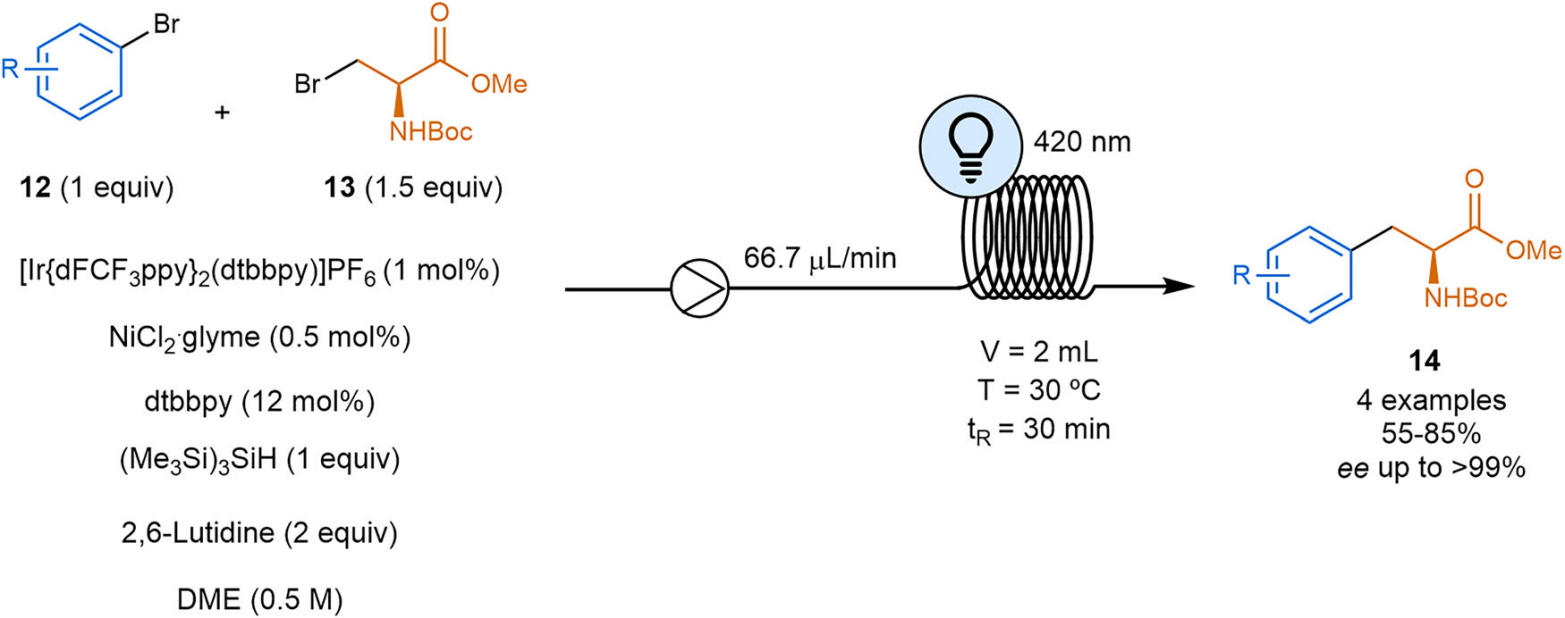
**Electrophilic cross-coupling of β-bromoalanine and aryl bromides**. This photocatalyzed process afforded unnatural amino acids **14**.

Unnatural amino acids **14** were also synthesized by means of metallaphotoredox process from β-bromoalanine derivatives **13** and different aryl bromides **12** (Scheme 4)^83^. Authors propose a silyl radical^84^ as a key player to generate the radical derived from the amino acid. The latest goes within the nickel catalytic cycle, being finally coupled to the aryl fragment in the final reductive elimination.

**Scheme 5:**
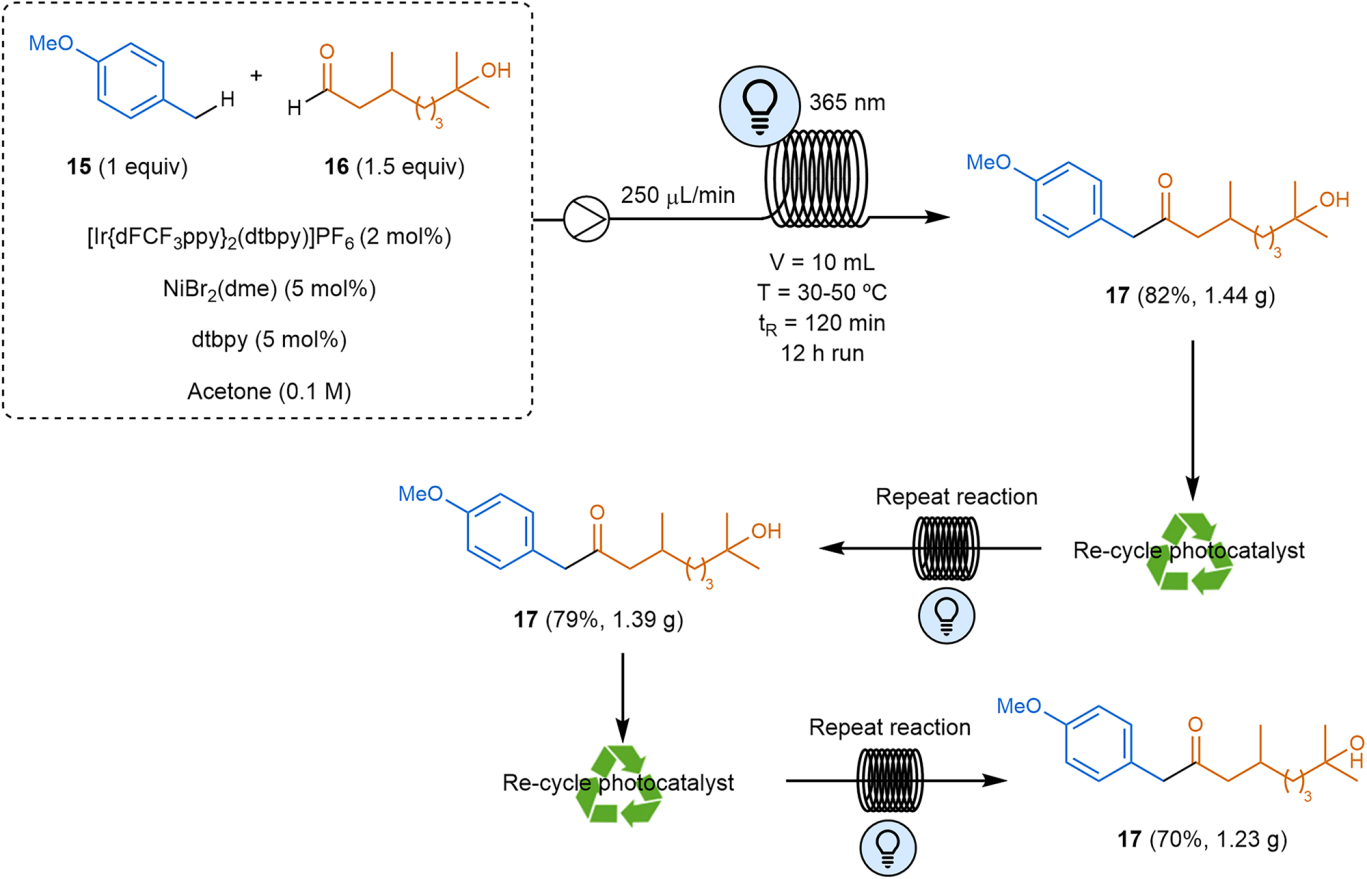
**Scalable photochemical process for the benzylation of aldehydes**. The reaction was optimized through efficient photocatalyst recycling and continuous high-yield production. Ppy: 2-phenylpyridine.

In a related transformation, Ley and coworkers reported the flow procedure of photoredox-catalyzed benzylic coupling of alkylarenes **15** to aldehydes **16** using an iridium photocatalyst together with a nickel co-catalyst^85^ based on a previous work developed in batch (Scheme 5)^86^. The authors developed a multigram-scale process using high-power LEDs and implemented a straightforward method for recovering and reusing the iridium photocatalyst via silica scavenger columns.

**Scheme 6:**
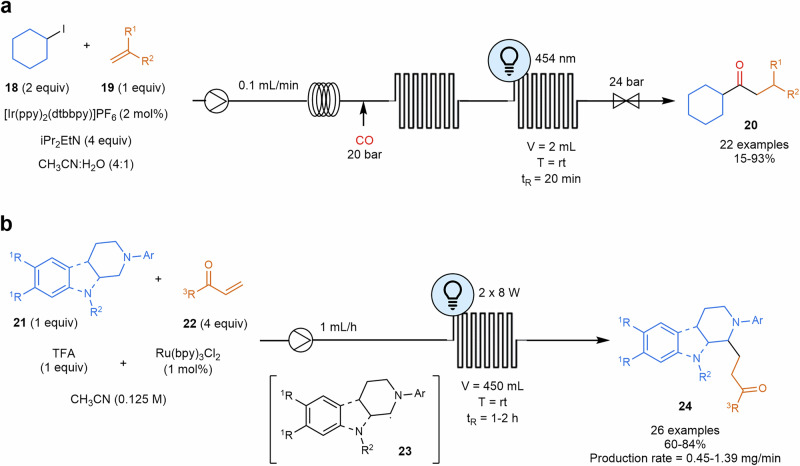
**Examples of photocatalyzed alkene transformations in flow. a** Carbonylative hydroacylation reaction of styrenes. **b** Photocatalyzed C(sp^3^)–H functionalization of N-aryl-protected tetrahydroisoquinolines and N-aryl-protected tetrahydro-β-carbolines via a-amino radical pathway.

Iridium photocatalysts have also been used in carbonylative alkene hydroacylation that works by the generation of a radical from unactivated alkyl iodides **18** and bromides that traps CO to form the acyl radical species. These acyl radicals react with alkenes **19**, leading to unsymmetrical ketones **20** (Scheme 6a). This multiphasic gas-liquid reaction can proceed successfully with a precise and controlled delivery of carbon monoxide due to the enhanced mass transfer in flow comparing with the batch process. Furthermore, the subsequent photochemical process can be more efficiently optimized^87^.

Some visible light promoted photoredox-catalyzed α-amino radical additions to Michael acceptors reactions in the presence of Brønsted acids have been reported. In this case, radical cations are formed by single-electron oxidation by the excited state of [Ru(bpy)_3_]^2+^. The subsequent deprotonation gives α-amino radical that would attack to the Michael acceptor. Under flow conditions, authors accessed a wide range of functionalized *N*-aryl-substituted tetrahydroisoquinolines and *N*-aryl-substituted tetrahydro-β-carbolines **24** (Scheme 6b)^88^. Access to 3,3-disubstituted dihydroquinoxalin-2(1*H*)-ones has also been exploited using a metal-free organic photoredox catalysis process, where unactivated alkyl bromides were the radical source to be introduced in substrates such as quinoxalin-2(1*H*)-ones^89^. The generation of α-amino radical from unprotected, aliphatic primary amines was reacted onto diethyl vinylphosphonate, leading to γ-aminophosphonates^90^. The translation from batch into a flow setup was successfully executed.

**Scheme 7:**
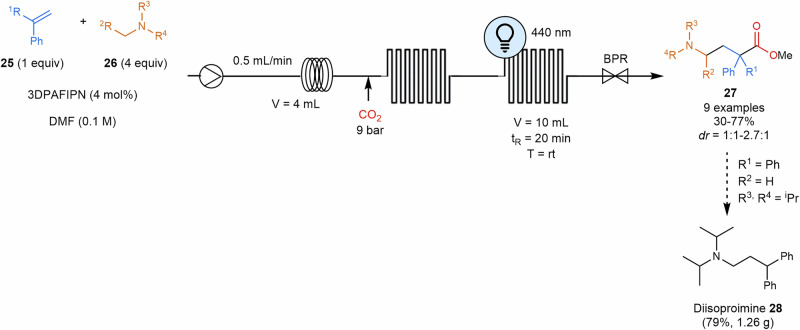
**Photocatalytic continuous flow aminocarboxylation**. 3DPAFIPN: 3,6-bis(diphenylamino)-9-(2,4,6-trifluorophenyl)isophthalonitrile. BPR back-pressure regulator.

Polyzos and coworkers have recently photochemically synthesized carbanions from diarylated alkenes. The aromatic alkenes serve as starting material to generate dystonic radical anions that perform nucleophilic reactions over a wide variety of substrates, leading to different transformations such as hydroalkoxylation, hydroamidation, aminoalkylation and carboxyaminoalkylation^91,92^. This elegant methodology presents an efficient alternative to traditional Grignard reagents. In this reaction, photochemically generated radical anions undergo nucleophilic attacks on weak electrophiles, leading to the formation of a second carbanion. This intermediate can be quenched by a proton source or reacted with a second electrophile, thereby expanding the chemical diversity. This approach reduces the waste of metal salts typically produced when using Grignard reagents, enhances safety, and eliminates the need for organic halides. Flow chemistry further enhances this process, enabling a reproducible and scalable photochemical hydroaminoalkylation towards diisoproimine **28** (1.26 g of product, 79% yield). The study showed a good tolerance with different amines **26** and unsubstituted aryl alkenes and α-alkyl styrene derivatives **25** were also compatible, leading to the subsequent products (Scheme 7).

**Scheme 8:**
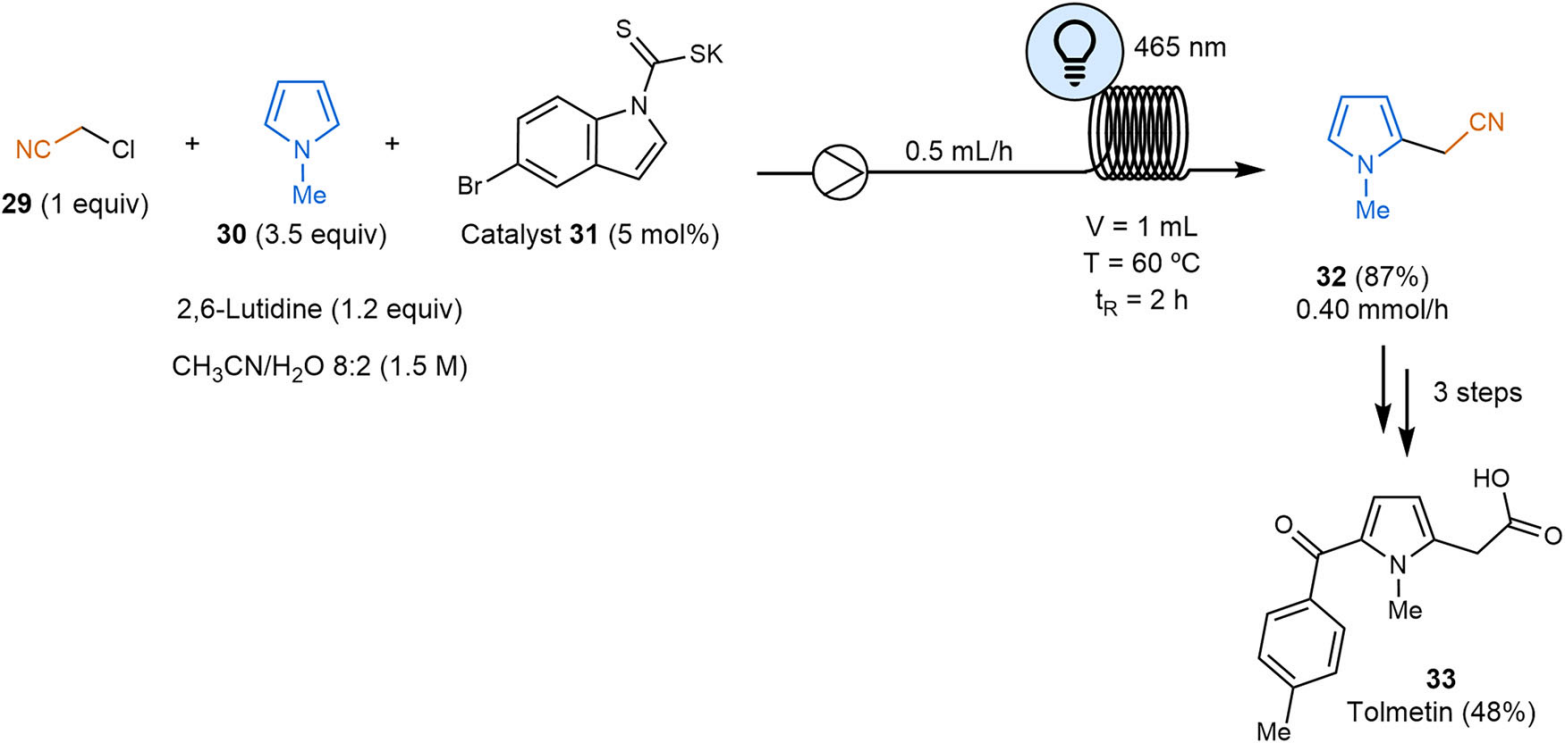
**Photocatalyzed continuous flow Giese-type radical conjugate addition**. The protocol was applied to the synthesis of intermediate **32** towards the marketed drug Tolmetin.

Melchiorre and coworkers used a rationale designed nucleophilic dithiocarbamate anion catalyst **31** that absorbs photons and activates benzyl and alkyl electrophiles via an S_N_2 pathway^93^. Following light absorption, the homolytic cleavage (C–S bond dissociation) generates open-shell intermediates that facilitate various radical transformations. As the authors argue, this strategy leverages a fundamental mechanistic process of ionic chemistry to access these open-shell species. Consequently, their efforts were directed toward the Giese-type radical conjugate addition to dimethyl fumarate, with one instance where they successfully translated their findings to a flow setup to synthesize 2-(1-methyl-1*H*-pyrrol-2-yl)acetonitrile **32**, an intermediate towards Tolmetin (**33**), a marketed nonsteroidal anti-inflammatory drug (Scheme 8).

#### C–H activation

**Scheme 9:**
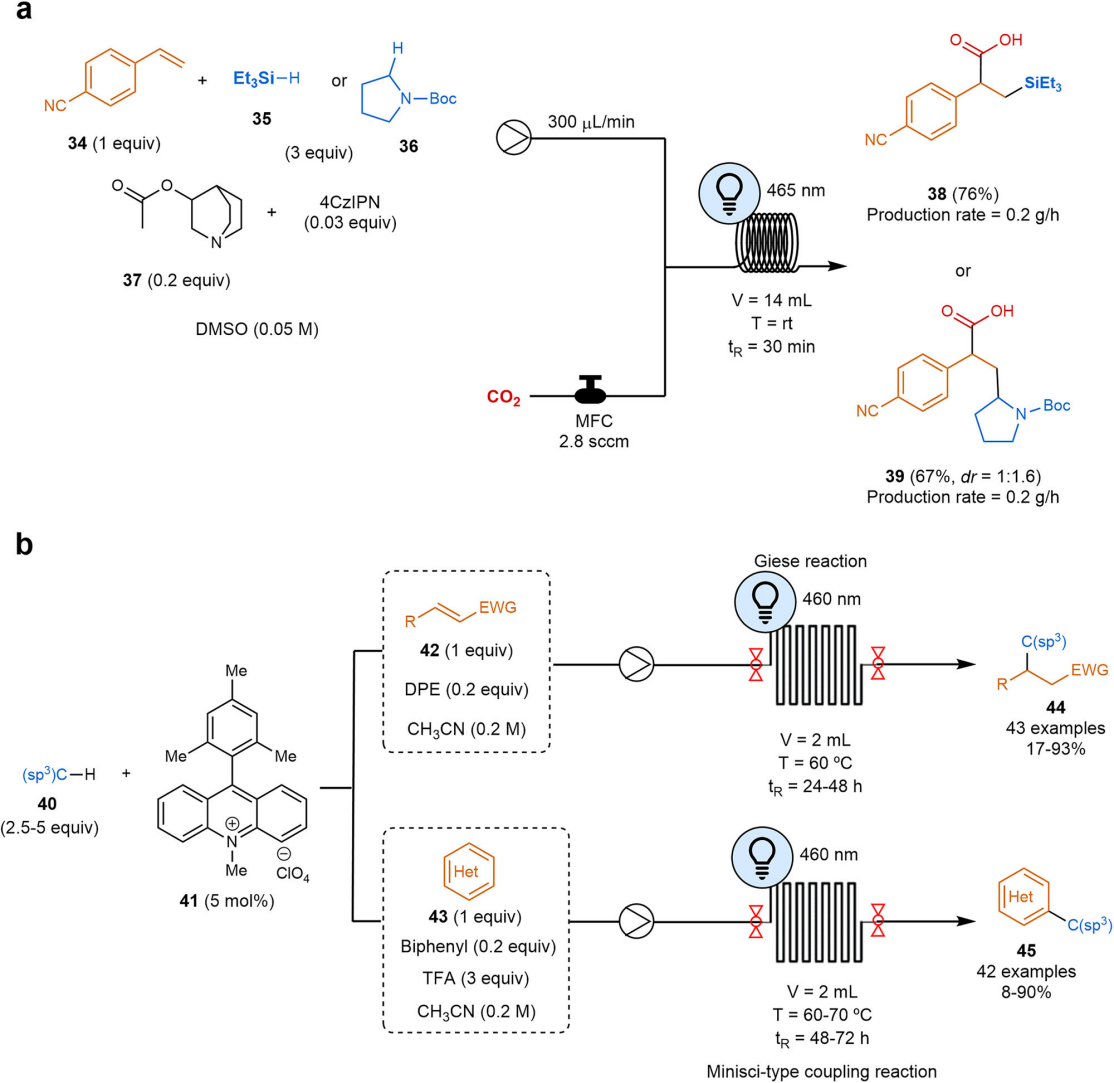
**Examples of photocatalyzed Si–H and C–H activation reactions in flow. a** Difunctionalization of alkenes via visible-light-mediated photoredox catalysis at gram scale. MFC mass flow controller. **b** Photocatalyzed Giese addition and Minisci-type cross-dehydrogenative coupling reaction. DPE diphenyl ether, EWG electron-withdrawing group.

Photomediated hydrogen atom transfer (HAT) catalysis consists of a new sustainable approach for C(sp^3^)–H and Si–H bond activation^94,95^. Wu and coworkers took advantage of the successful generation of radical species through visible-light-mediated photoredox catalysis to perform difunctionalization of alkenes^96^. Silacarboxylation and carboxylation were elegantly performed, creating a radical through a hydrogen atom transfer that would subsequently attack an olefin, generating a new radical species that is photochemically reduced to a carbanion. These anionic species would react with electrophiles such as carbon dioxide, leading to the difunctionalization of olefins. This work draws inspiration from the previous studies by Martín and Yu, which focus on the photocatalytic functionalization of styrenes with carbon dioxide using photocatalytic transition metal-based systems. However, these reaction systems typically require the use of transition metals, strong bases or prefunctionalized radical precursors^97,98^. In the context of flow chemistry, this process was successfully scaled up to the gram scale of compounds **38** and **39**, benefiting from the enhanced light penetration (Scheme 9a). Additionally, it leverages the advantages of pressurized microtubing reactors, which improve gas/liquid mixing efficiency and facilitate the use of gaseous reactants^47,62^. In terms of scalability, photocatalytic reactions involving gases are still under development, for instance, the numbering-up strategy reported by Noël and coworkers for the aerobic oxidation of thiols to disulfides using oxygen^99^.

In a recent work, authors used stopped-flow microtubing reactors as ideal systems to screen and optimize variables in the photochemical efficient activation of non-activated C–H bonds, especially primary C(sp^3^)–H bonds, via Giese additions and Minisci-type cross-dehydrogenative coupling reactions (Scheme 9b)^100^. Acridinium-based photocatalysts **41**, redox mediators like diphenyl ether or biphenyl, together with trace chloride anions, displayed remarkable HAT catalysis capabilities. The optimized conditions found in the stopped-flow microtubing reactor were later efficiently translated into a scaled-up flow synthesis. Within this topic, hydrogen atom transfer catalysis has also been used for mild monofluoroalkenylation of C–H bonds with *gem*-difluoroalkenes through a bromine radical that abstracts the hydrogen atom from a C(sp^3^)–H bond to form the alkyl radical^101^. This alkyl radical would merge with a fluoroalkenyl radical to form the final product. In this case, the use of a continuous flow setup reduces the irradiation time, leading to high yields. In the other case, the C–H activation of a C(sp^2^) was performed classically by Pd(II)/Pd(IV) at elevated temperatures. However, merging this catalytic cycle with a visible-light photoredox catalysis has enabled to perform acylation with a wide range of aldehydes over indols at room temperature^102^. Reduction time and increase in yields were obtained using flow chemistry.

**Scheme 10:**
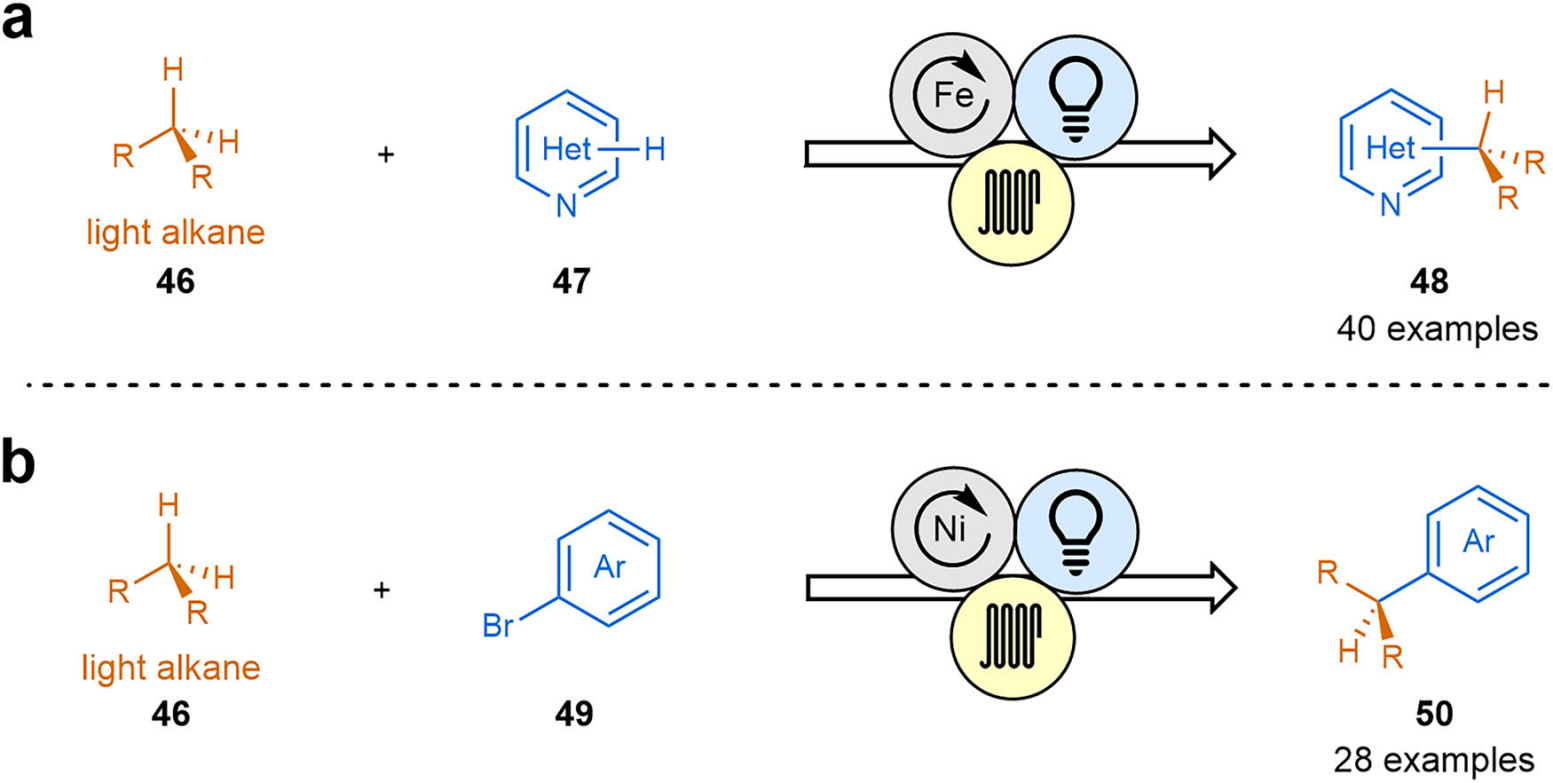
**Transformations of gaseous alkanes via HAT photocatalysis. a** Alkylation of heteroarenes by Minisci reaction employing light alkanes. **b** First cross-coupling reaction between gaseous alkanes and aryl bromides.

In a great contribution, Nöel and coworkers have used HAT catalysis to perform heteroarene alkylation via Minisci reactions using C1–C4 gaseous alkanes **46** under mild conditions^103^. In this case, the authors propose that a photo-induced iron-catalyzed ligand-to-metal charge transfer could be employed to cleave the strong C(sp^3^)–H bonds of C1–C4 alkanes **46** and perform photocatalytic alkylation of heteroarenes **47** (Scheme 10a). This work is particularly significant as it demonstrates the use of inexpensive, carbon-based gaseous feedstocks to introduce short alkyl groups into a variety of heteroarene rings. The results underscore the value of flow technologies in enabling the controlled use of gaseous alkanes as alkylating agents, offering precise regulation of gas equivalents for efficient and selective transformations. This work is the follow-up of a contribution where these authors also applied successfully the incorporation of light gaseous alkanes to heteroaryl bromides **49** via HAT photocatalysis combined with nickel catalysis^104^, being the first cross-coupling reaction involving gaseous alkanes (Scheme 10b).

**Scheme 11:**
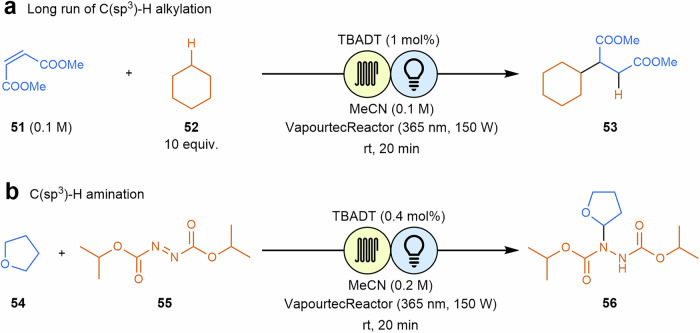
**An example where OSN enables efficient use of TBADT as HAT photocatalyst. a** Application to alkylation reactions**. b** Application to amination reactions.

The decatungstate anion is discussed to be the most versatile HAT photocatalyst, finding application in different transformations in flow, such as alkylation^105,106^, acylation^107^, amination^108–110^, and oxygenation^111,112^. Nevertheless, these HAT transformations are still not widely applied in industrial processes due to catalyst costs. One interesting example was developed by Nöel and coworkers, where organic solvent nanofiltration (OSN) was used for the recovery of the tetrabutylammonium decatungstate (TBADT) catalyst^113^. C(sp^3^)–H bond functionalization reactions (alkylation and amination transformations) were carried out and a SolSep BV NF030306 membrane which permitted satisfactory recovery of both catalyst and product, applying two consecutive OSN steps (Scheme 11). Remarkably, TBADT recovery had no detrimental effect on reaction performance, as the yields obtained with this inline OSN system were comparable to those obtained with pristine TBADT.

Single-electron transfer (SET) oxidations of C(sp^3^)–H bonds were also nicely executed in continuous setups, leading to a widening of the chemical space. 1,2,3,4-Tetrahydroisoquinoline (THIQ) is a good example, as several transformations have been studied over the carbon adjacent to the nitrogen group. Stephenson and coworkers performed the C–H oxidation of THIQs using super-stoichiometric amounts of BrCCl_3_ as a terminal oxidant^114^, while the subsequent nucleophilic reaction, cyanation, among others, was performed in batch. Zeitler used a microreactor in the photoredox functionalization of THIQs to generate aza-Henry products^115^. Filipovic performed Mannich, cyanation or alkynylation transformations under flow conditions with a simple and readily available Ru catalyst^88^. Rueping and coworkers also reported several photoredox functionalizations of THIQs in flow using Rose Bengal as a photoredox sensitizer^116^.

#### Photocycloadditions

**Fig. 3:**
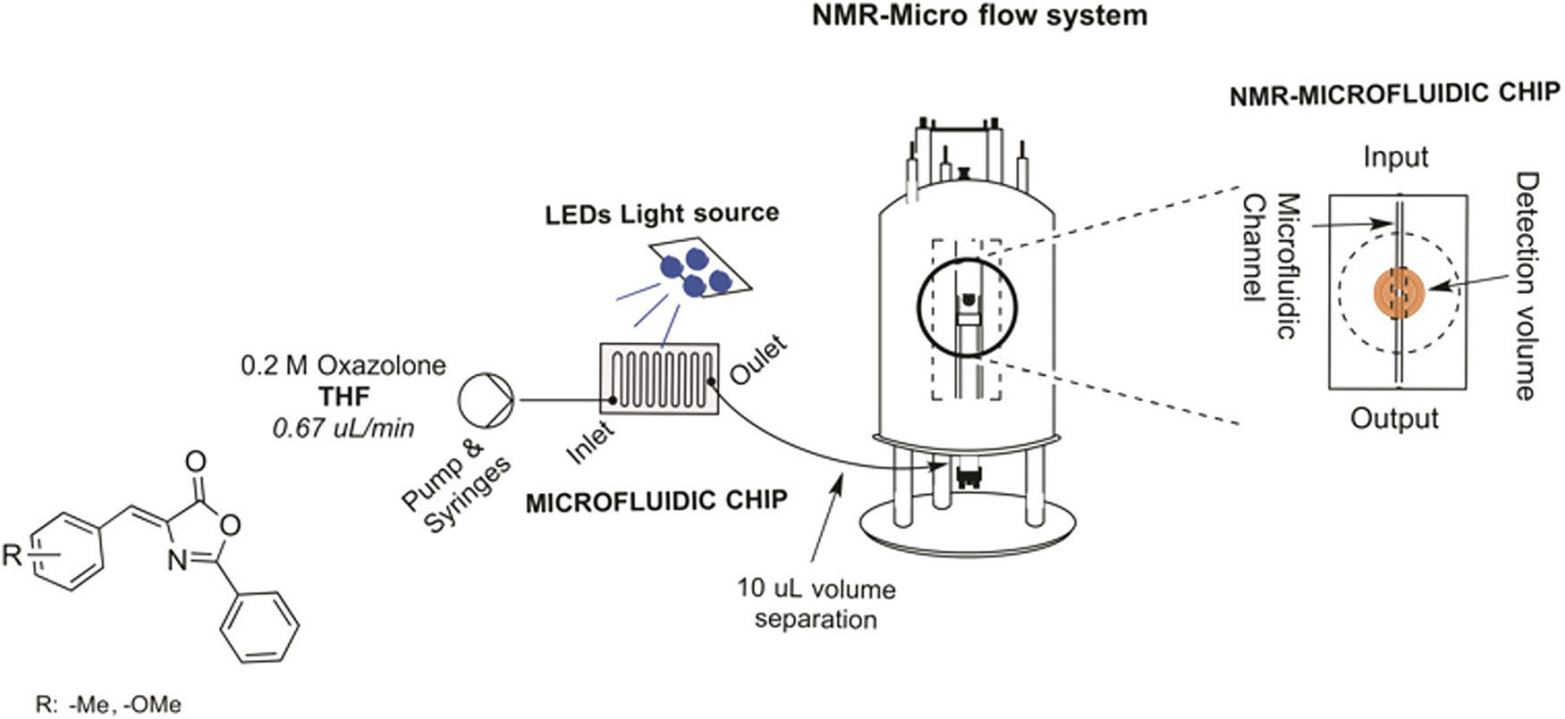
Microphoto-NMR setup. Reprinted from García-Montero, A.; Rodriguez, A. M.; Juan, A.; Velders, A. H.; Denisi, A.; Jiménez-Osés, G.; Gómez-Bengoa, E.; Cativiela, C.; Gómez, M. V.; Urriolabeitia, E. P. Metal-free [2+2]-photocycloaddition of (Z)-4-aryliden-5(4H)-oxazolones as straightforward synthesis of 1,3-diaminotruxillic acid precursors: synthetic scope and mechanistic studies. ACS Sustain. Chem. Eng., 5 (9), 8370–8381, Copyright (2017), with permission from American Chemical Society.

The use of flow chemistry has also benefited [2+2] photocycloaddition reactions between two olefins to form cyclobutanes. This attractive transformation implies a rapid assembly of complex molecular structures with high regio- and stereoselectivity. Beeler and coworkers designed a flow photochemical platform with a cone reactor that significantly improved the [2+2] photocycloaddition of cinnamate derivatives run under UV light. The reaction was performed using a bis(thiourea) to template the two molecules of alkene (cinnamate) to facilitate dimerization, increasing the conversion and diastereoselectivity. Palladium acetate has also served to template (*Z*)-2-aryl-4-aryliden-5(4*H*)-oxazolones. Because of the palladium constraint, the exocyclic C=C bonds of the oxazolones are placed face-to-face in proximity, in an optimal arrangement to undergo a [2+2]-photocycloaddition^117,118^. The reaction was accelerated using microreactors in continuous flow, with the added advantage of scaling up to produce larger quantities of compounds. In addition to improved process efficiency, the adoption of inline analytical techniques has accelerated reaction optimization. In a subsequent study, inline nuclear magnetic resonance (NMR) enabled optimization of the process variables and real-time reaction monitoring. By coupling a microreactor with a planar microcoil detector chip, inline monitoring of the continuous flow, photo-assisted reaction significantly reduced reaction times to just 30 min, yielding a pronounced product distribution of four distinct isomers (Fig. 3). Moreover, the reaction was continuously analyzed, providing valuable insights into its progress throughout the entire reaction time. This marked the first use of inline NMR to monitor a photochemical reaction in a flow setup^18,119^.

The use of an organophotoredox catalyst allowed to perform a fully stereoselective synthesis of cyclobutanes in solution in the absence of templates. In this respect, authors demonstrated how methyl esters of 1,2-diaminotruxinic bis-amino acids can be obtained as single isomers with complete regio- and stereoselectivity by Ru-photocatalyzed [2+2] photocycloaddition of (*Z*)-4-aryliden-5(4*H*)-oxazolones in solution. The reaction was performed under blue light, in batch and flow reactors, with a much better performance being achieved in flow devices (reaction time of 1 h vs 24 or 48 h)^120^. Radical cation-initiated dimerization of electron-rich alkenes rendering cyclobutanes had also been performed^121^. In this study, the authors conducted a comparative analysis of batch versus flow synthesis, demonstrating a reduction in catalyst loading and reaction times when using a flow setup. They applied the optimized conditions to achieve a scalable synthesis of the styrene-based lignan dimeric natural product magnosalin.

#### Halogenation of organic compounds

**Scheme 12:**
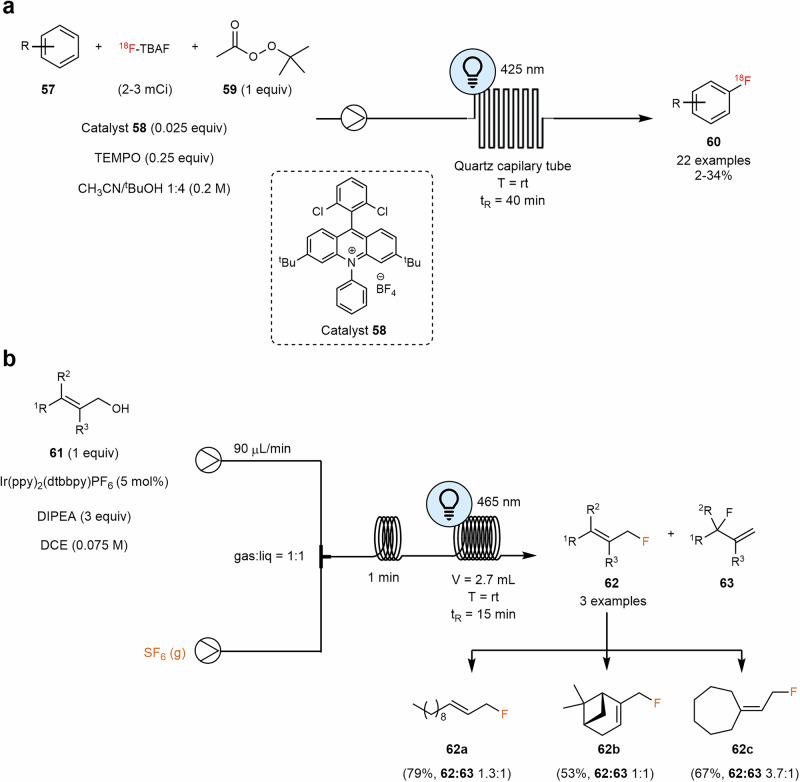
**Examples of photoredox fluorination of organic compounds. a** Direct radiofluorination of arene C–H bonds via photoredox catalysis. **b** Photoredox deoxyfluorination using SF_6_.

Visible-light halogenation reactions have been reviewed recently^122^. Since the first reports of benzylic brominations under visible light and no catalyst^123,124^, several catalytic reactions for halogenation under visible light irradiation were recently developed. These reactions are usually performed under milder conditions, allowing to avoid high temperatures and non-selective UV light. Direct C–H radiofluorination catalyzed by organic photo-oxidation catalysts under mild reaction conditions was reported by Nicewiz, Li and coworkers. ^18^FNBu_4_, acridinium catalyst **58**, TEMPO and blue LEDs (425 nm) were employed to generate radio-labeled fluorobenzenes^125^. In this work authors updated a previous work^126^ using *tert*-butyl peroxyacetate **59** as an oxidant instead of oxygen, blue LEDs instead of laser irradiation and flow chemistry instead of batch (Scheme 12a). Good results were obtained even replacing the LEDs by laser (20.2%, compared with 23.5% yield, using a 3.5 W laser). The approach also relied on SET oxidation of an aromatic compound **57** to generate the corresponding radical cation, which then reacts with a fluoride anion. This process is effective with both electron-rich and electron-poor aromatic systems, heterocycles, and bioactive molecules. It is particularly well-suited for late-stage transformations of complex molecules, enabling the production of tracers for positron emission tomography.

In 2016, Jamison reported the use of safe, inexpensive, yet inert SF₆ for deoxygenative fluorination. Under blue LED irradiation and in the presence of an iridium catalyst, allylic alcohols **61** were converted to allyl fluorides **62** in moderate yields. Notably, performing the reaction under continuous flow conditions led to improved yields (Scheme 12b). The authors postulate that this improvement was likely due to the increased pressure, mass transfer, and interfacial area of the continuous-flow system^127^.

Gómez et al. also explored dehalogenation reactions, developing a novel instrument based on integrated fiber optics for real-time, in situ NMR monitoring of the reductive dehalogenation of α-bromoacetophenone on a nanoliter scale. This was achieved by irradiating the NMR chip with 525 nm LEDs and performing the reaction in stopped-flow mode. The system is capable of monitoring reactions in small NMR detection volumes of 25 nL while providing uniform irradiation through various low-power light sources with high photon flux. In contrast to traditional NMR setups, which often encounter challenges like excessive irradiative heating and significant light intensity decay when attempting uniform UV-vis illumination, this system offers a more efficient solution^128^.

#### Trifluoromethylation and related transformations

**Scheme 13:**
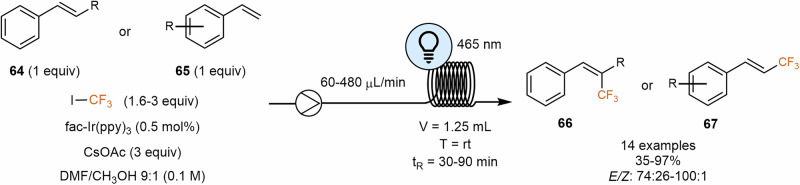
**Trifluoromethylation of terminal and α- or β-substituted styrenes via visible light photoredox catalysis**. In this flow protocol, CF_3_I is used as an inexpensive trifluoromethylation reagent.

Trifluoromethylation of C–H bonds is highly sought after due to the crucial role of the CF_3_ group in the pharmaceutical and agrochemical industries. Various research groups have focused on developing methodologies that photochemically generate the trifluoromethyl radical, often incorporating flow chemistry to enhance efficiency and scalability^129–138^. In one of these works, Noël and colleagues^135^ were able to introduce successfully the trifluoromethyl group in styrenes **64** or **65** in spite of the unique challenges with regard to unproductive polymerization, product isomerization, oxidation, dimerization and nucleophilic trapping of the starting materials. In this work, they reported a facile method for both trifluoromethylation and hydrotrifluoromethylation of terminal and α- or β-substituted styrenes **64** or **65** via visible light photoredox catalysis using CF_3_I as an inexpensive trifluoromethylation reagent (Scheme 13).

In batch reactions, the *E/Z* ratio was often poor. The authors hypothesized that the thermodynamically most stable E-isomer was initially formed and then converted into the *Z*-isomer via an energy transfer (ET) mechanism. During their investigations, they observed that the *E/Z* ratio decreased with longer reaction times. Continuous flow photomicroreactors enable the photocatalytic trifluoromethylation of styrenes, achieving complete conversion with a high *E/Z* ratio due to shorter reaction times and more efficient irradiation. These flow reactors are particularly well-suited for photochemical transformations, as they ensure homogeneous irradiation of the reaction mixture and allow for easy scale-up through numbering up. The reaction was completed in less than an hour in flow, resulting in excellent *E/Z* ratios, thereby preventing isomerization via the ET mechanism. These findings were consistently observed along different substrates. Notably, a significant acceleration and higher yields were also achieved when the reaction was conducted in the photomicroreactor, reducing the reaction time from 24-72 h in batch to just 0.5–1.5 h in flow.

#### Other transformations

**Fig. 4:**
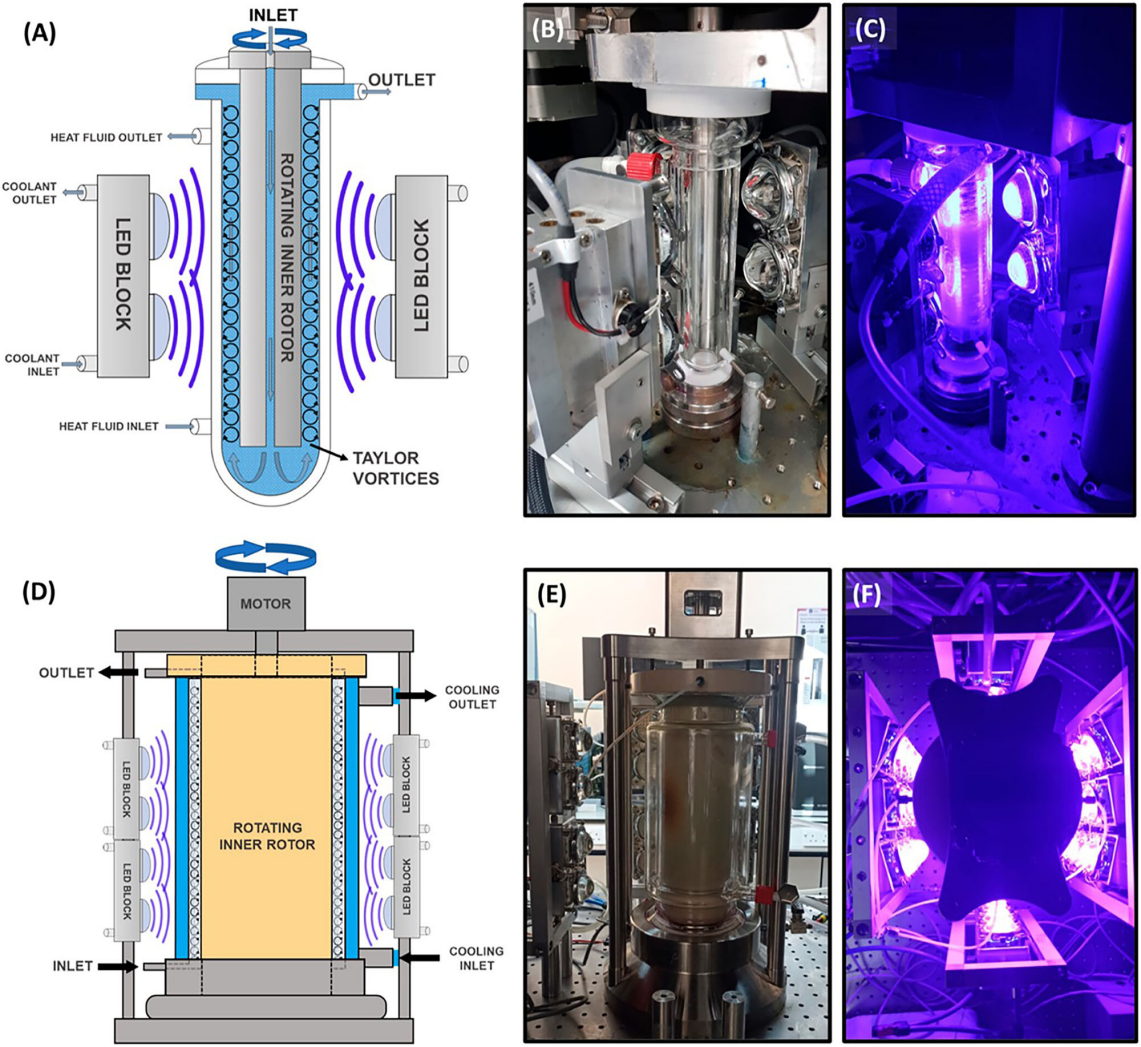
Schematic views and photographs of the PhotoVortex and the scaled-up PhotoVortex. **A** PhotoVortex. **D** Scaled-up PhotoVortex. **B** Photograph of the PhotoVortex showing the jacket, rotor and the modified 410 nm high-powered LEDs (600W, 3 × 200W blocks) with LEDs turned off and **C** LEDs turned on. **E** Photograph of the large PhotoVortex showing the jacket, rotor and half of the modified 410 nm high-powered LEDs (3 kW, 15 × 200W blocks). **F** Photograph of the large PhotoVortex taken from directly above when LEDs are turned on to illustrate the configuration of the LEDs around the reactor. "Reprinted from Teixeira, R. I.; Clarke, T. H. W.; Love, A.; Sun, X.-Z.; Kayal, S.; George, M. W. Scale-up of continuous metallaphotoredox catalyzed C–O coupling to a 10 kg-scale using small footprint photochemical Taylor vortex flow reactors. Org. Process Res. Dev., 29 (1), 34–47, Copyright (2025), with permission from American Chemical Society. License CC BY 4.0 (https://creativecommons.org/licenses/by/4.0/)".

C–O coupling has also been accomplished via dual metallaphotoredox catalysis, providing a mild and efficient approach for synthesizing alkyl-aryl ethers. MacMillan and coworkers showed that a combination of an iridium photocatalyst and a nickel cocatalyst could effectively promote C–O bond formation between aryl bromides and primary or secondary alcohols, yielding alkyl-aryl ethers under mild conditions with good functional group tolerance^139^. Recently, George and coworkers applied a modified version of the previously reported photochemical Taylor Vortex Flow Reactor (PhotoVortex), where Taylor vortices and a short irradiated path length enable efficient photochemical reactions through excellent mixing (Fig. 4)^140^. In a small PhotoVortex (8 mL irradiated volume), productivities around 1 kg/day and >10 kg/day in a large PhotoVortex (185 mL irradiated volume) were achieved, with good product yields (>90%) and low catalyst loadings (0.1 to 0.5 mol% of [Ir{dF(CF_3_)ppy}_2_dtbbpy]PF_6_), enabled by excellent mixing ensuring sufficient mass transfer between short-lived photoexcited and other transient specie^140^. This work presents a strategy for recovering the costly iridium catalyst via column chromatographic purification. The authors also suggest alternative methods, including the heterogenization of the catalyst^141–143^ and organic solvent nanofiltration^113,144,145^. Molecules smaller than the membrane’s molecular weight cut-off (MWCO) pass through, while larger species are selectively retained. In this context, Kappe and colleagues enlarged a [Ru(bpy)_3_]^2+^ complex by incorporating it into a polyamidoamine (PAMAM) dendrimer-based macromolecule. This macrocatalyst was successfully tested and recycled multiple times across various metallaphotoredox transformations^144^.

**Scheme 14:**
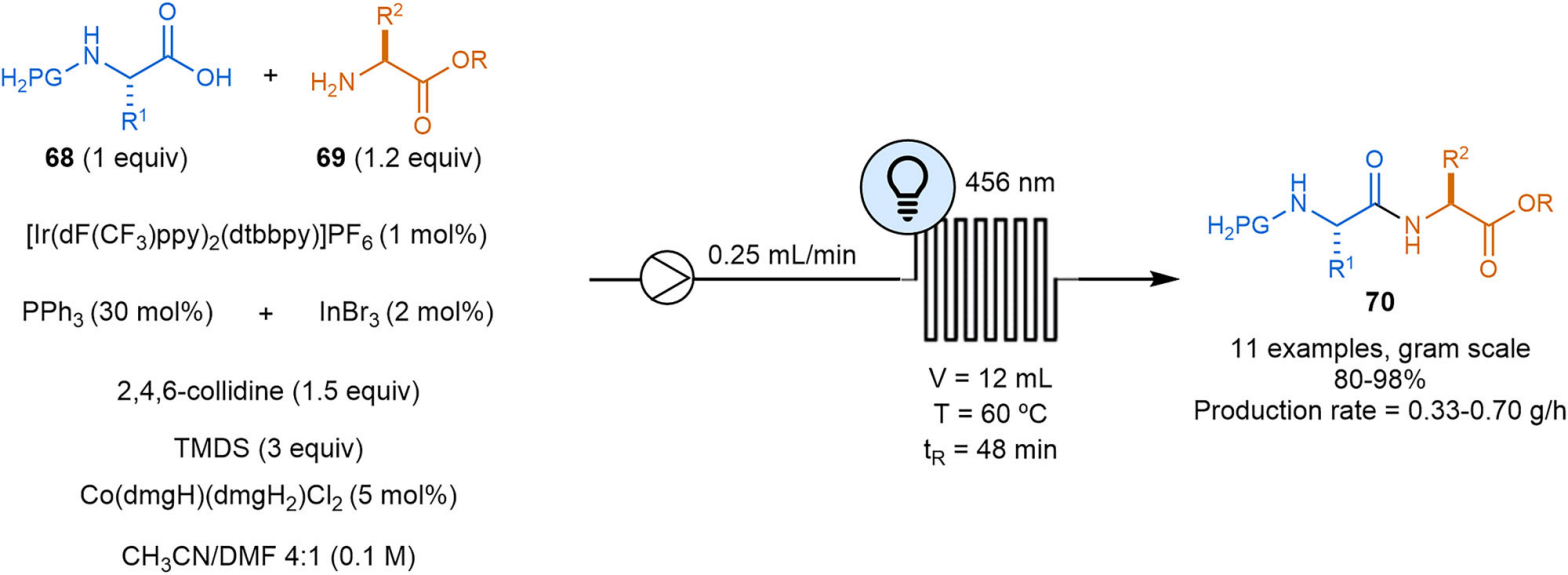
**Photoredox gram-scale synthesis of dipeptide derivatives**. dmgH: dimethylglyoximate monoanionic; dmgH_2_: neutral dimethylglyoxime.

Dipeptide formation^146^ has been successfully achieved through synergistic photoredox, cobaloxime, and organophosphorus triple homogeneous catalysis. In this approach, the catalytic cycle was initiated by the single-electron oxidation of PPh_3_ using a visible light-excited photocatalyst. The resulting phosphine radical cation reacts with a carboxylate to form a phosphoranyl radical. A subsequent single-electron transfer between this radical and a cobaloxime catalyst, Co(III), facilitates the formation of the dipeptides **70** via aminolysis, while generating a phosphine oxide that is recycled through reduction with sacrificial silanes and catalytic InBr_3_. The use of a flow setup enhances the photoredox step, leading to higher yields (Scheme 14).

One of the most notable advances in photochemical reactor technology is the spinning disc reactor (SDR), particularly the rotor-stator spinning disc reactor (pRS-SDR). This design generates high shear forces and turbulence, producing thin liquid films (typically 12–76 µm) on a rapidly rotating disc^147–149^. Such films not only maximize light penetration and irradiation efficiency but also ensure excellent mixing and superior mass and heat transfer. A key advantage of SDRs is their ability to effectively handle suspensions containing solids without clogging^150,151^, while also facilitating milder operating conditions (e.g., room temperature instead of cryogenics) for reactions that typically require extreme environments. While batch reactors might initially outperform SDRs for clear solutions, the low surface-area-to-volume ratios limit light penetration at scale, as dictated by the Beer-Lambert law.

Beyond SDRs, other mechanically agitated reactors are employed in photochemistry, including vortex reactors^45,152^, photovap systems^153,154^, oscillatory flow reactors^152^, ultrasonic photochemical reactors^155,156^ or continuous stirred-tank reactors^157,158^. More common in process intensification are capillary/tubular reactors^47^, which overcome limited light penetrations in batch by creating narrow channels or employing highly efficient mixing, thereby enhancing mass, heat, and photon transfer^45,148,154,159^. These systems improve safety for hazardous reactions by minimizing reactor hold-up^160^ and they enable "novel process windows" including operations at elevated temperatures or pressures^161^. Such innovations are transforming chemical manufacturing across diverse applications^47,162^.

Future efforts must be focused on developing more robust and easily recyclable homogeneous and heterogeneous photocatalysis, as well as reactor designs that maximize light–reactor interaction through optimized geometries and integrating energy-efficient light sources like specific UV-B/C LEDs^152^. Solar light as a free and sustainable energy source remains a key ambition, requiring strategies to manage its inherent variability^31,48,148,163^. Finally, rigorous modeling of mass, heat and photon transport phenomena will be essential to ensure the confident and widespread industrial adoption of flow photochemistry^152^.

### Electrochemistry in flow

Chemical synthesis mediated by electrolysis (electrosynthesis) presents a promising alternative to traditional methods and operates under mild conditions, offers atomic efficiency, and is environmentally friendly^42–44,164–168^. Unlike conventional processes, it avoids the use of harsh reducing or oxidizing agents that generate exothermic reactions and are frequently highly polluting. Moreover, electrolysis is synthetically valuable because it can enable new reaction mechanisms and promote reactions with high chemo- and regioselectivity^169^. The renewed interest in electrolysis-based chemical synthesis stems from the growing demand for more sustainable chemical processes. By utilizing electrons as reactants, electrolysis enhances atomic economy and minimizes waste. Additionally, these selective processes eliminate the need for derivatization or protection/deprotection reactions. This field significantly benefits from applying continuous flow processes to electrolytic syntheses due to increased mass transport and accelerated reaction rates^33,170–173^. Furthermore, electrochemistry in flow offers the advantage of integrating in situ analytical tools in the reactor that allow real-time monitoring of reaction evolution^18,119^.

When direct electrolysis fails to achieve the desired selectivity or yield, an indirect approach using a redox catalyst—also known as a mediator (Med) or electron carrier—is often employed. This mediator plays a crucial role in facilitating electron transfer and driving the reaction forward. This process follows the principles formalized by Steckhan in the 1980s^174,175^. For effective indirect electrocatalysis, mediators must meet several key criteria^164^: they should be recyclable, stable in both oxidation states, and capable of efficient electron transfer between the electrode and the reactant. Additionally, they should exhibit fast electrochemical kinetics and be easily separable from the final products. Mediators can participate in either a single-step or a cascade of homogeneous processes, ultimately leading to the target product. This enables the continuous recycling of redox couples, making the system more sustainable. Beyond improving selectivity, electrocatalysis lowers the redox potential required for functional group transformations and significantly accelerates reaction rates^43,44,176^.

The resurgence of organic electrosynthesis is largely driven by advances in electrochemical flow technology. Electrochemical flow reactors can be categorized as divided or undivided cell reactors, depending on the presence or absence of a physical separator between electrodes. Divided reactors prevent mixing of anodic and cathodic products and are desirable to avoid side products in highly selective reactions. In contrast, undivided reactors share the reaction medium, simplifying the design, although with a lack of selectivity. Beyond this classification, parallel flow reactors consist of two closely spaced parallel electrodes (<1 mm apart) through which the reactant solution flows. This parallel-plate configuration minimizes electrolyte resistance—proportional to the interelectrode distance—allowing for higher currents and reduced concentrations of supporting electrolyte, improving efficiency and enhances the sustainability of the chemical process.

Most reported synthetic electrochemistry relies on reactions occurring at a single electrode, with by-products generated at the counter electrode^51^. As a result, the desired electrochemical transformations are typically either oxidative or reductive. In contrast, redox-neutral electrochemistry—also known as paired or coupled electrosynthesis—enables simultaneous and desirable half-electrode reactions at both electrodes, improving material and energy efficiency. Despite these advantages, it remains relatively underdeveloped, and the potential of flow chemistry in organic electrosynthesis has been demonstrated^51^. In conventional paired electrochemical setups, a major challenge lies in matching the generation and interelectrode transport rates of different highly reactive intermediates. These mismatches often lead to undesired side reactions. However, microfluidic electrochemical systems offer precise control and rapid transport of reactive species within micrometer-scale channels, improving reaction performance due to the higher surface-to-volume ratios. A microfluidic redox-neutral electrochemistry platform with broad applicability to SET chemistry was introduced, including radical-radical cross-coupling, Minisci-type reactions, and nickel-catalyzed C(sp^2^)–O cross-coupling. The extremely thin interelectrode gap in the flow cell allows for rapid diffusion that outpaces radical decomposition, selectively yielding the desired cross-coupling products. Notably, their study found that increasing the interelectrode distance significantly reduced cross-coupling yields. Among various applications, two reactions required the presence of homogeneous catalysts (mediators). Preactivated carboxylic acid *N*-hydroxyphthalimide (NHP) esters have been utilized in photocatalyzed Minisci-type reactions to functionalize heterocycles. This strategy circumvents the high oxidation potential required for direct carboxylic acid activation, thereby improving functional group tolerance^177,178^. Additionally, this platform also facilitated nickel-catalyzed C(sp^2^)–O cross-coupling, yielding O-aryl esters.

#### Transition metal-catalyzed free reactions

**Scheme 15:**
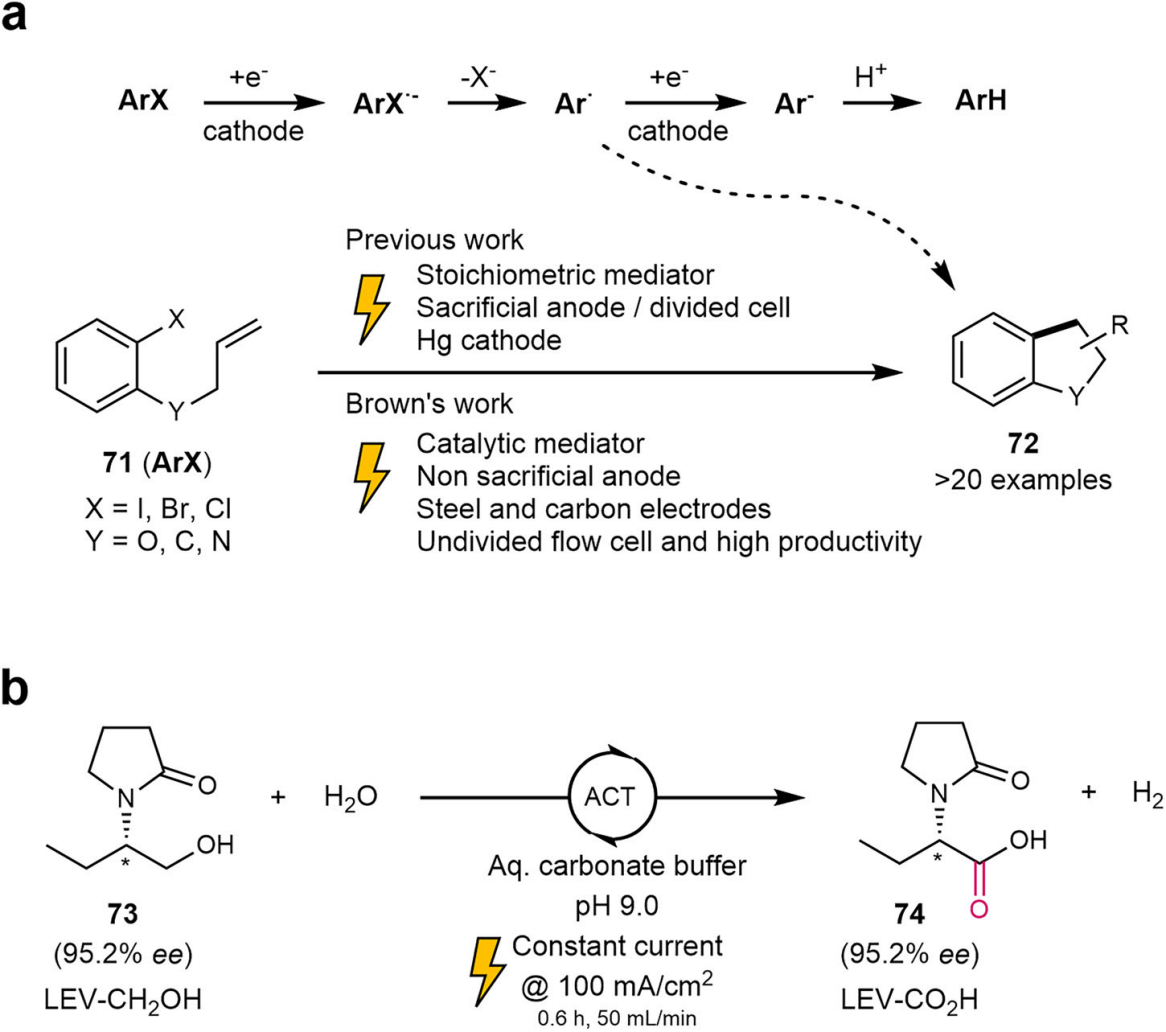
**Examples of transition metal-catalyzed free electrochemical reactions in flow. a** Cathodic radical cyclizations of aryl halides in continuous flow. **b** Synthesis of a Levetiracetam precursor. LEV levetiracetam.

Cathodic radical cyclizations of aryl halides **71** have been accomplished in an undivided flow electrolysis cell using phenanthrene as a mediator at loadings as low as 0.05 equivalents, without the need for a sacrificial anode. It is proposed that the reaction proceeds via mediated homogeneous electron transfer occurring in a reaction layer separated from the cathode, which explains the observed selectivity^179^. The mediator is effective in sub-stoichiometric amounts (0.05 equivalents), enhancing the practicality of cathodic radical cyclization. The methodology applies to O-, N-, and C-tethered substrates, yielding bicyclic, tricyclic fused and spirocycle systems (**72**). In these examples, the presence of a strongly reducing mediator favors radical formation instead of the hydrogenolysis of the C–X bond that takes place in the absence of the mediator (Scheme 15a).

Related to the creation of spirocycles, initial efforts are currently being developed to perform electrochemical oxidative decarboxylation of malonic acid derivatives. These substrates, after decarboxylation and acetal formation with methanol, undergo a double-ring closure under acidic conditions, yielding diverse spiroketals^180^.

The synthesis of precursor **74** of the antiepileptic drug Levetiracetam has also been explored by Stahl’s group^181^. The reaction was carried out in water with a buffer of NaHCO_3_/Na_2_CO_3_ and a 5 mol% of the catalyst 4-acetamido-2,2,6,6-tetramethylpiperidin-1-oxy (ACT) as redox mediator. The reaction in batch took 11 h with a 92% yield and 92% of *ee*. An important decrease in time was observed when the reaction was performed in an undivided cell (1.2 h) and in a divided cell (0.6 h) under continuous flow (Scheme 15b). In addition, the enantiomeric excess increased up to 99% maintaining the good yields. The reaction was scaled up to 200 g, in which hydrogen was the sole stoichiometric byproduct.

When larger electrode surfaces are required to improve the reaction outcome at medium scale, new electrochemical flow reactors have been described to overcome some challenges related to heat and mass-transfer limitations. For instance, a rotating cylinder reactor was used by Kappe and coworkers in collaboration with MSD for a Hofmann rearrangement using NaBr as mediator. The interelectrode distance was the same as in the small batch reactor, and the amount of material and time to scale-up the reaction was reduced^160^.

#### Transition metal-catalyzed transformations

**Scheme 16:**
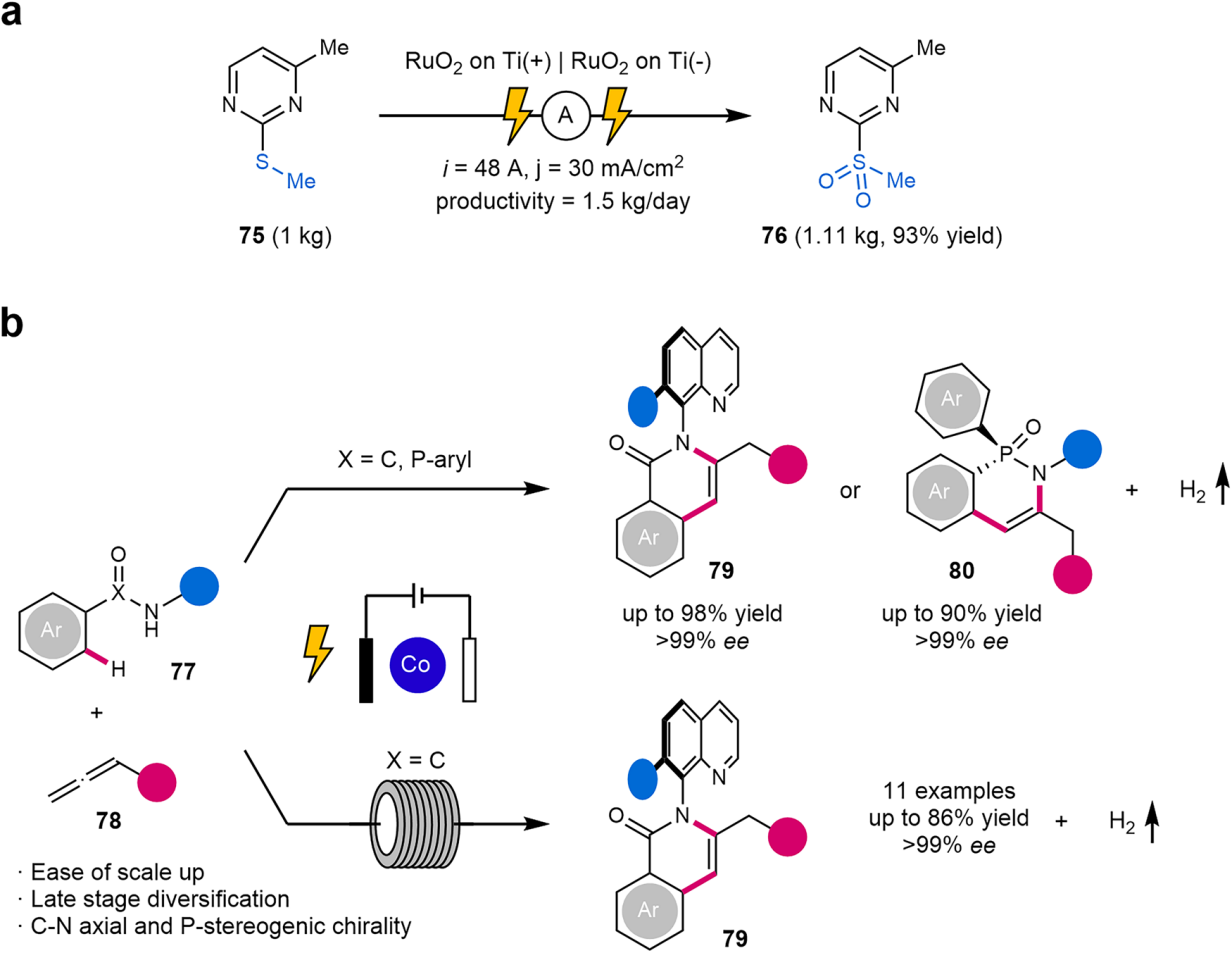
**Some transition metal-catalyzed electrochemical transformations in flow. a** Electrochemical oxidation of thioesthers to sulfones. **b** Atropochiral C–H bond activation for the enantioselective annulation of allenes.

Another electrochemical methodology oriented to kilogram scale was described by Bottecchia and Lehnherr in 2022^182^. RuO_2_ on Ti electrodes promoted the electrochemical oxidation of thioesthers **75** to sulfones **76**, of interest for APIs development, avoiding the use of toxic and more expensive oxidizing agents for this type of chemical modification. Continuous flow allowed a productivity of 1.5 kg/day, in which this work is supposed to be one of the first examples reported on such a scale in the pharmaceutical industry (Scheme 16a).

Cobalt was also a suitable metal to carry out electrochemical transformations. In this regard, Ackermann reported an atropochiral C–H bond activation for the enantioselective annulation of allenes **78** in both batch and flow protocols^183^. Complex organic C–N axially chiral molecules **79** and *P*-stereogenic compounds **80** were prepared in very good yields and enantioselectivities even for the preparation of biologically relevant molecules such as cholesterol, menthol, probenecid and tamibarotene (Scheme 16b). The electrochemical flow reaction was suitable to produce more than 9 g of one enantiomeric product in a 68% yield and 99% of *ee*.

**Fig. 5:**
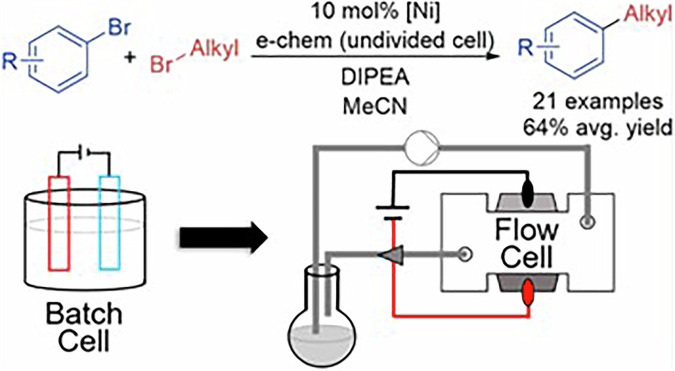
Electrochemical nickel-catalyzed C(sp^2^)-C(sp^3^) cross-coupling reaction. "Reprinted from Franke, M. C.; Longley, V. R.; Rafiee, M.; Stahl, S. S.; Hansen, E. C.; Weix. Zinc-free, scalable reductive cross-electrophile coupling driven by electrochemistry in an undivided cell. ACS Catal., 12 (20), 12617–12626, Copyright (2022), with permission from American Chemical Society”.

Attending to Nickel, Hansen and Weix reported in 2022 an electrochemical C(sp^2^)–C(sp^3^) cross-electrophile coupling reaction between aryl and alkyl bromides without using Zn sources^184^. In this work, NiBr_2_ was used as catalyst and bipyridines as ligands to promote the reaction in an undivided batch cell with a Ni cathode and graphite anode (Fig. 5). After the preparation of more than 20 products in batch, the reaction was optimized in an electrochemical flow system to be scaled-up to 4 g by using two flow cells in parallel, obtaining a yield of 73% when the current density was 5.7 mA/cm^2^.

**Scheme 17:**
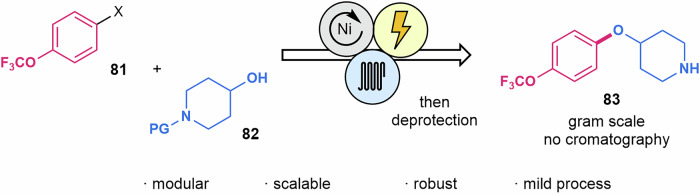
**Construction of C–O bonds by means of Nickel-mediated flow electrocatalysis**. This methodology was applied to the synthesis of an intermediate of delamanid.

The formation of aryl-alkyl ether bonds through cross-coupling of alcohols with aryl halides represents a useful strategic departure from classical S_N_2 methods^185^. An electrochemical arylation catalyzed by Ni complexes has been recently reported by Kappe and Laudadio in continuous flow^186^. A recirculation mode through an undivided cell was more effective than the single pass and using an Ar atmosphere. This approach allowed the formation of C–O bonds for 11 different examples. Using aryl halide **81** and piperidyl alcohol **82**, the method was suitable for the scalable formation of compound **83**, a key intermediate of delamanid, an API for the treatment of multidrug-resistant tuberculosis (Scheme 17).

Nickel electrocatalysis has also been applied successfully under flow conditions to enable a mild and scalable methodology to perform cyclopropanation of alkenes using bulk chemical dichloromethane and other widely available *gem*-dichloroalkanes as methylene precursors^187^.

In the last years, the combination of photochemistry and electrochemistry has also been possible to activate challenging substrates and create novel synthetic methodologies^188^. The integration of electrophotocatalysis in continuous flow systems has also been described, showcasing the benefits of these technologies.

**Scheme 18:**
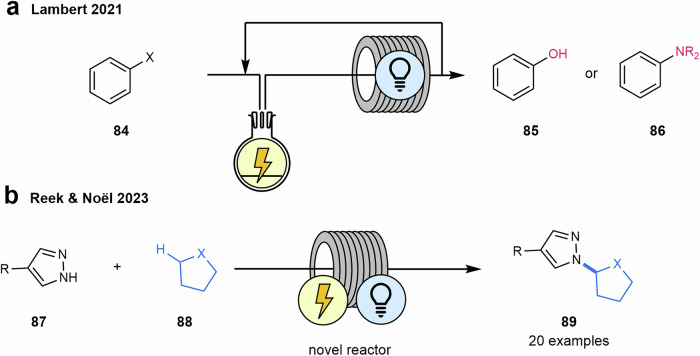
**Examples of electrophotocatalytic reactions in flow. a** Electrophotocatalytic C–H Heterofunctionalization of arenes. **b** Electrophotocatalytic heteroarylation.

Photoredox reactions are typically initiated by the single-electron oxidation of the arene ring and are often performed with relatively electron-rich substrates due to their lower oxidation potentials. In contrast, achieving such reactions with weakly electron-deficient or electron-neutral arenes, such as those bearing halogen atoms or benzene, has proven more challenging. Huang and Lambert efficiently carried out the heterofunctionalization of arenes **84**, including hydroxylation, alkoxylation, and amination reactions, using 2,3-dichloro-5,6-dicyano-1,4-benzoquinone (DDQ) as an electrophotocatalyst. Taking advantage of the fact that the photochemical step in these reactions proceeds at a slower rate than the electrochemical one, they successfully translated the batch process into a continuous setup. By coupling a small electrochemical cell with one or more photochemical chambers, they succeeded in the synthesis of phenols **85** and amines **86** (Scheme 18a)^189^.

**Fig. 6:**
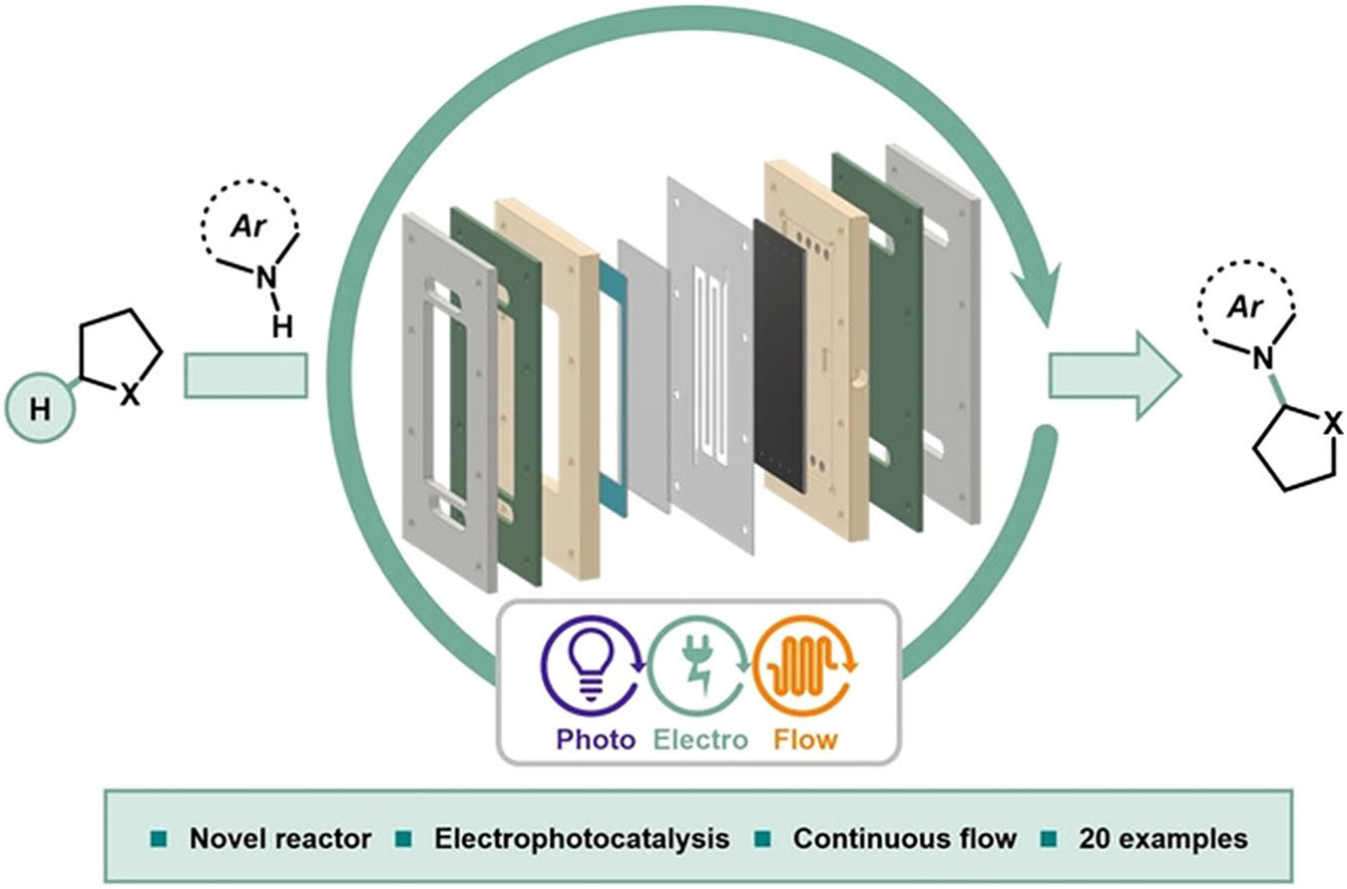
Flow reactor for electophotocatalysis. **“**Reprinted from Ioannou, D. I.; Capaldo, L.; Sanramat, J.; Reek, J. N. H.; Noël, T. Accelerated electrophotocatalytic C(sp^3^)–H heteroarylation enabled by an efficient continuous-flow reactor. Angew. Chem. Int. Ed., 2023, 62, e202315881. Copyright (2023), with permission from John Wiley-VCH GmbH”.

In the same context, Noël and colleagues introduced an innovative flow reactor concept for electrophotocatalysis (EPC) that harnesses both photons and electrons simultaneously (Fig. 6)^190^. Their design features a transparent electrode and utilizes cost-effective materials, making the technology accessible and efficient.

They demonstrated the reactor's capabilities by developing an efficient electrophotocatalytic heteroarylation of C(sp³)–H bonds of compounds **88** (Scheme 18b). The authors highlighted that the same setup also enables purely electrochemical and photochemical transformations. The key to achieving this transformation lies in the integration of HAT via ligand-to-metal charge transfer (LMCT) photocatalysis and electrochemical oxidation. This novel synthetic approach for forming C(sp³)–N bonds in flow merged electrochemically induced radical-polar crossover with HAT photocatalysis, operating under exceptionally mild conditions. The electrophotocatalyzed heteroarylation occurred at room temperature and delivered short reaction times, significantly improving productivity over existing methods for the synthesis of the coupling products **89**.

Similarly, Nöel and coworkers recently took advantage of the electrophotocatalysis to perform oxidant-free amidation of aldehydes at room temperature and using an inexpensive and abundant Fe(III) catalyst^191^.

Attending to reactor design, significant advancements have addressed the broad applicability of flow electrocatalysis. Spinning electrode reactors (RCEr), for example, provide excellent mixing and mass transport even at very low flow rates, enabling the seamless processing of slurries without clogging or fouling and facilitating the rapid detachment of gas bubbles from electrode surfaces^50,160^. This design allows for seamless scale-up from milligram to multi-kilogram quantities without re-optimization of reaction conditions, simply by maintaining a consistent interelectrode gap. Beyond RCErs, parallel plate flow reactors remain popular due to their simplicity, modularity, and cost-effectiveness, with 3D printing enabling rapid, customizable, and low-cost prototyping^192,193^. For demanding reactions, cylindrical "pipe cells" offer superior pressure resistance, operating at high temperatures and pressures^194^.

The field is further propelled by the integration of high-throughput experimentation (HTE) and automation. Automated electrochemical flow platforms facilitate rapid data collection, reaction optimization via Design of Experiments (DoE), and library synthesis with minimal human intervention and reagent consumption^50,195^.

### Automation in flow

Increasing productivity in the preparation of organic molecules is a key factor for synthetic chemists. The chances of discovering new compounds of interest are dependent on how fast experiments can be performed and how the methodology is able to create molecular diversity. Traditional approaches used to achieve this goal have been combinatorial chemistry or parallelization techniques, which allow to improve the efficiency of synthetic processes, reducing both cost and time. From a medicinal chemistry point of view, HTE has been widely considered to improve experimental frequency. With this technique, many reaction conditions can be screened at the same time to find suitable reaction parameters and/or even execute the synthesis of compound libraries at low scale, providing a vast number of organic scaffolds readily for biochemical assays.

Automated platforms are often described as high-throughput due to the continuous operation mode and their capacity for real-time optimization. These features offer unique opportunities for the optimization of reaction parameters (e.g., temperature, residence time, stoichiometry, concentration) with minimal material consumption. However, reactions are carried out sequentially, and the number of experiments per unit of time is limited. In contrast, batch HTE offers the chance of evaluating large datasets of discrete reaction variables for rapid screening (e.g., catalysis, ligands, solvents). Both approaches are complementary, as batch HTE is appropriate for hit identification across broad condition spaces, whereas flow-based self-optimization is very useful for fine optimization.

The combination of HTE with continuous flow chemistry allowed the creation of fully automatized systems for reaction optimization, library synthesis or multistep reaction protocols. In this manner, the efficiency and robustness of the platform decrease the cost and time to perform the synthesis. In addition, novel synthetic methodologies can also be performed in an automated mode^195^. PAT can also be integrated, reducing human intervention and allowing the generation of large datasets to control reaction outcomes or monitoring the steady-state^25^. For instance, inline flow infrared (IR), UV-VIS or Raman spectroscopy, mass spectrometry (MS) or NMR analysis have been widely implemented^196–198^. The use of PAT tools has also been integrated in photocatalytic organic synthesis^199^. Moreover, computer-aided synthesis planning (CASP), based on decision-making algorithms, can also be adapted to these automated systems to propose synthetic plans, retrosynthetic analysis or condition recommendations, in which the design of the next experiment takes place autonomously in closed-loop flow experiments^54,55^. The synthesis of a vast number of molecules assisted by computers and analyzed by analytical systems can also be complemented with the integration of biological platforms to extract real-time data of the process, creating full automated and integrated systems from the own design-synthesis-analysis cycle to the obtention of biochemical data^196,197^.

Most of the chemical transformations reported during the last years in automated systems involved non-catalytic reactions (amide bond formation, functional group protection or deprotection, condensations, reductions, S_N_Ar…) to facilitate following steps in telescoped reactions^198,200–202^, and even with the implementation of artificial intelligence^203^. The integration of biological platforms has been more investigated in heterogeneous protocols by using packed-bed reactors or even in work-up steps to increase the purity of the molecules^197^. Despite the well-known advantages of the previous approaches, homogeneous catalysis presents an outstanding alternative in the preparation of challenging scaffolds, and some recent methodologies have been described under continuous flow using this technology, mainly for library synthesis and/or reaction optimization.

**Scheme 19:**
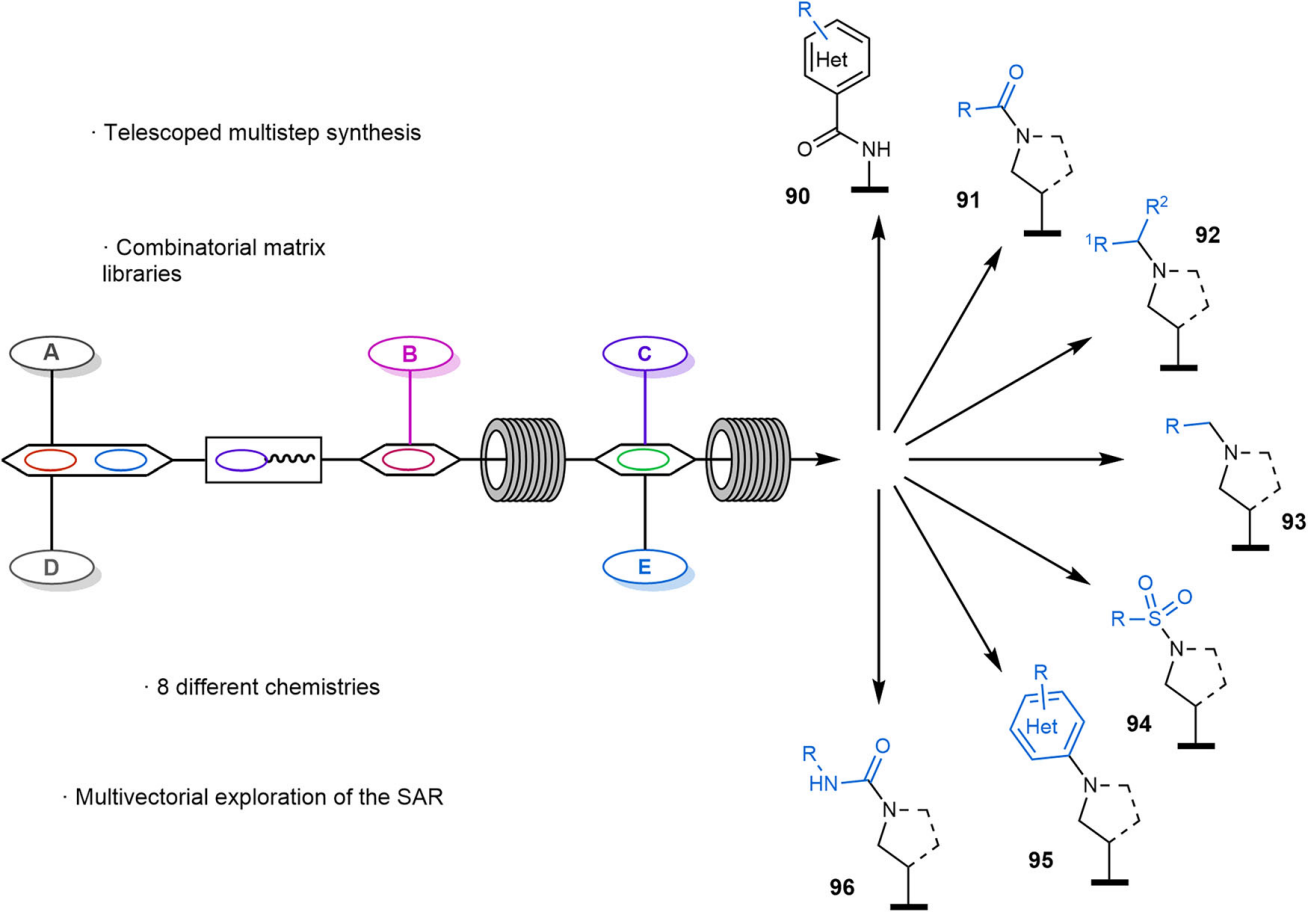
**Synthesis of compound libraries using 8 different synthetic methodologies**. This work was focused on accelerating drug discovery by quickly accessing a broad range of chemical diversity.

First, the preparation of compound libraries is highly desirable to increase chemical diversity, as a vast number of organic molecules can be prepared to explore new chemical space. In 2024, Alcázar reported an automated flow protocol for multistep library synthesis oriented to drug discovery for a rapid exploration of the chemical space^204^. In this work, up to 8 different synthetic methodologies could be carried out in continuous flow involving established methodologies, metal-catalyzed transformations and modern photoredox reactions in a homogeneous version (Scheme 19). First, amide bond formation followed by a Negishi coupling reaction allowed the preparation of 48 compounds (**90**) with a productivity of 4 products per hour, with a success rate of 96%. *N*,*N*-Dimethylformamide (DMF) was used as carrier solvent to control the dispersion of the system and avoid cross-contamination between reaction slugs. Then, a mix of iridium and nickel-catalyzed photochemical transformation was incorporated into the reaction sequence through ART reaction to achieve C(sp^2^)–C(sp^3^) bond formation reactions. With a success rate of 88%, 24 products were synthesized in 24 h (**91**). The nucleophilicity of the free amines obtained under this protocol could also be used in subsequent transformations such as amide couplings (**91**), reductive aminations (**92**), alkylations (**93**), sulfonamide (**94**) or S_N_Ar (**95**) and urea (**96**) formations. A broad range of chemical diversity was generated, which is crucial to accelerate drug discovery programs, and high-throughput purification (HTP) was used to isolate the compounds and obtain representative quantities for biological assays to generate structure-analysis-relationships (SAR) data^205^.

In addition to the synthesis of libraries, some recent developments include reaction optimization approaches, as many experimental time and chemicals are required to explore reaction parameters. Thus, HTE has been implemented in microfluidic platforms to carry out different reaction conditions and facilitate automation processes, improving reproducibility and reducing material consumption. To achieve a real-time optimization, the integration of all parameters into a computer gives rise to self-optimization systems, which provide an autonomous method for data-enriched reaction development^206,207^. For instance, Jensen and coworkers combined an automated flow platform with mixed-integer nonlinear programming (MINLP) algorithm to optimize the catalyst turnover number with eight catalyst candidates in a Suzuki–Miyaura cross-coupling reaction between a 3-chloropyridine and 2-fluoropyridine-3-boronic acid pinacol ester^208^. Only 40 μL of reaction solution was used for each automated flow experiment, and the algorithm required just 60 experiments to achieve optimum conditions.

**Scheme 20:**
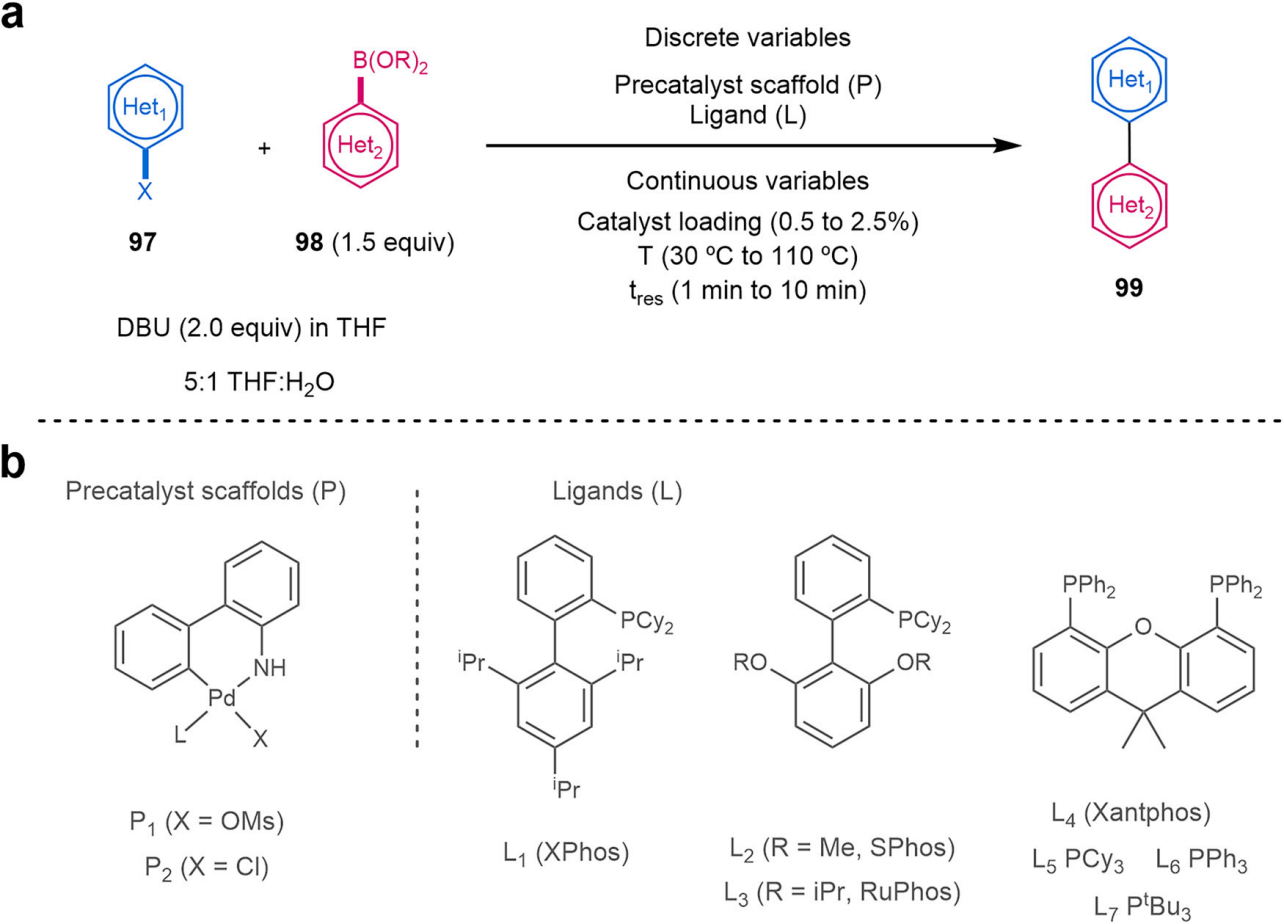
**Example of the optimization of a Suzuki–Miyaura cross-coupling reaction in flow. a** Reaction conditions, relying on the use of the base 1,8-diazabicyclo[5.4.0]undec-7-ene (DBU). **b** Precatalyst scaffolds and ligands used for this reaction optimization.

**Fig. 7:**
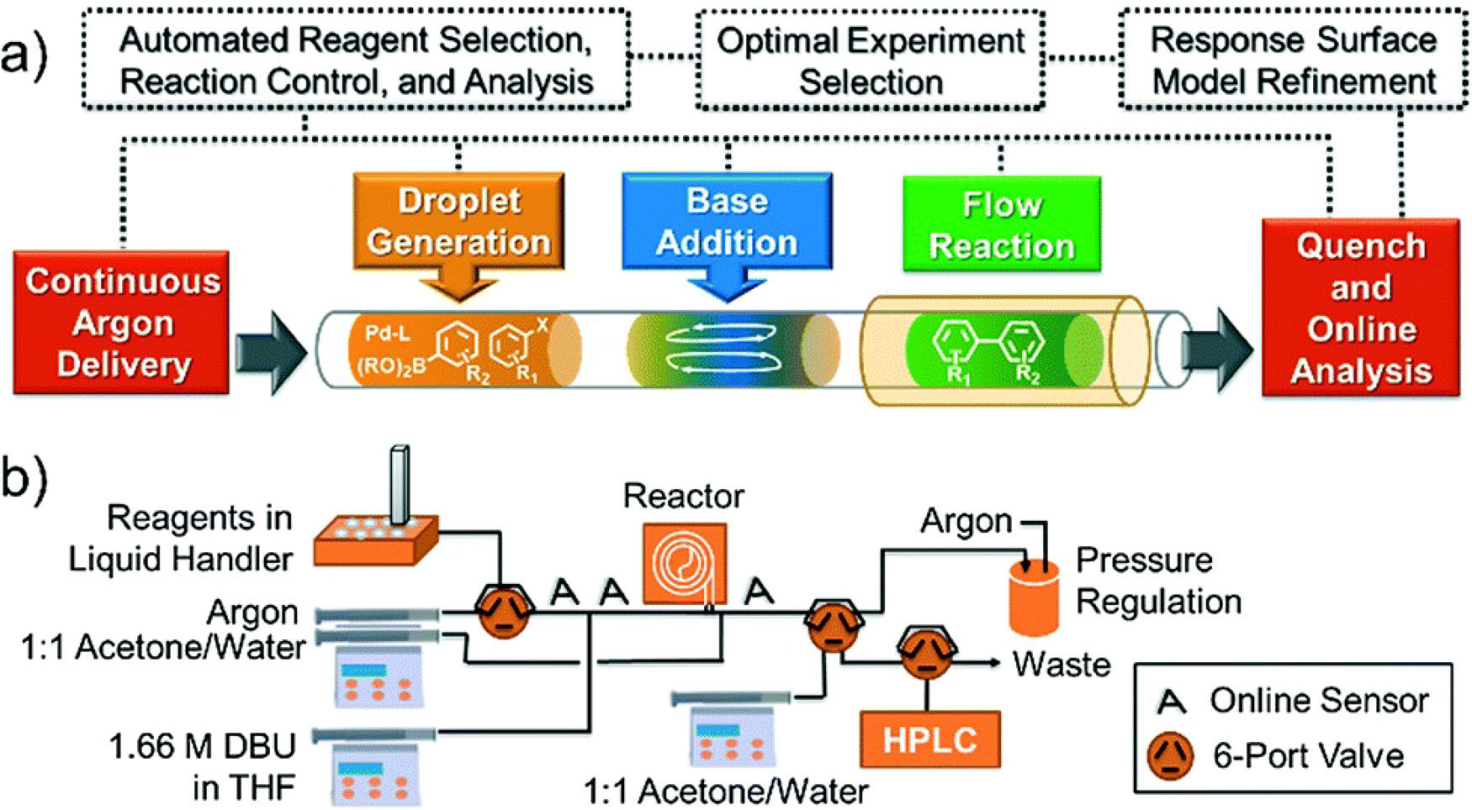
Concept and flow diagram for automated Suzuki–Miyaura cross-coupling optimization. **a** General concept of the project. **b** Flow diagram for automated Suzuki–Miyaura cross-coupling optimization. "Reprinted from Reizman, B. J.; Wang, Y. M.; Buchwald, S. L.; Jensen, K. F, Suzuki–Miyaura cross-coupling optimization enabled by automated feedback. React. Chem. Eng. 1 (6), 658–666, Copyright (2016), with permission from American Chemical Society. License CC BY 4.0 (https://creativecommons.org/licenses/by/4.0/)".

Due to the high importance of the Suzuki–Miyaura cross-coupling in organic chemistry and pharmaceutical industry, Jensen and Buchwald have also described another optimization of discrete variables (palladacycle and ligand) and continuous variables (temperature, loading and reaction time) for this coupling reaction simultaneously in continuous flow (Scheme 20, Fig. 7). This complex optimization could be done within 96 experiments for a more rational selection of the reaction parameters for heterocyclic systems. One precatalyst scaffold (**P1**) and XPhos ligand (**L1**) were suitable in most cases. However, a substantial improvement of the reaction outcome was observed if the ligand was adapted to match the rates of the different steps of the catalytic cycles (selecting from precatalysts **P1** or **P2**, and ligands **L1** to **L7**)^209^.

**Fig. 8:**
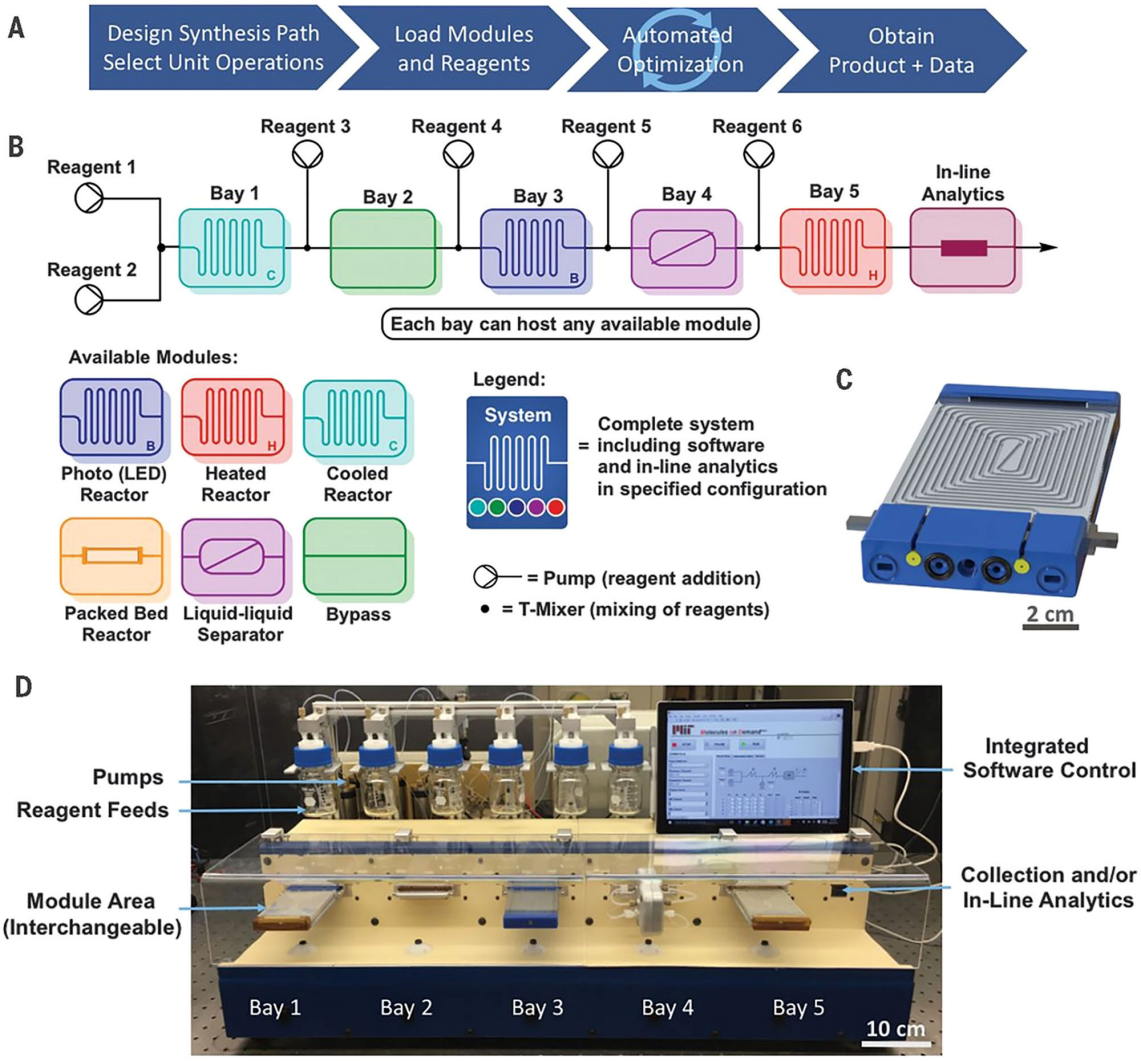
Continuous-flow chemical synthesis reconfigurable system. **A** General four-step protocol for using the system. **B** Representative configuration of the components in the system. **C** CAD (computer-aided design) representation of the LED reactor; shown is a view of the end that attaches to a universal bay on the system. See Figs. S2, S4, and S5 for details of the fluidic and electrical connections in the universal bay. **D** Schematic representation of the configuration shown in (**B**) and available modules. "Reprinted from Bédard, A.-C.; Adamo, A.; Aroh, K. C.; Russell, M. G.; Bedermann, A. A.; Torosian, J.; Yue, B.; Jensen, K. F.; Jamison, T. F. Reconfigurable system for automated optimization of diverse chemical reactions. Science, 361, 1220–1225, Copyright (2018), with permission from American Association for the Advancement of Science. License 1648477-2”.

The research teams led by Jensen and Jamison collaborated to develop a highly adaptable system aimed at streamlining chemical experimentation. This integrated platform combined hardware components for synthesis and purification, analytical tools for reaction monitoring, and a user interface with software-based control (Fig. 8). This configuration enabled automated optimization, exploration of reaction scopes, and scale-up procedures for six key transformations in organic chemistry: Buchwald-Hartwig amination, Horner-Wadsworth-Emmons olefination, reductive amination, Suzuki–Miyaura cross-coupling, nucleophilic aromatic substitution (S_N_Ar), and a visible-light photoredox reaction. The continuous-flow setup proved effective for these synthetic methodologies, facilitating the preparation of over 50 high-yield products, predominantly with autonomous operation and consequently, a reduced manual input^210^.

Another example was reported by Pfizer researchers, combining HTE with flow chemistry and liquid chromatography-mass spectrometry (LC-MS) analysis in the same automated platform. They were able to obtain data points from 5760 combinations of the Suzuki–Miyaura cross-coupling between quinoline and indazole coupling derivatives, by screening 12 ligands, 8 bases and 4 aqueous-based solvents. Then, 1500 coupling reactions at the nanomolar scale could be done in just 24 h, showcasing the benefits of merging HTE with continuous flow in reaction optimization and synthesis of libraries^211^.

**Fig. 9:**
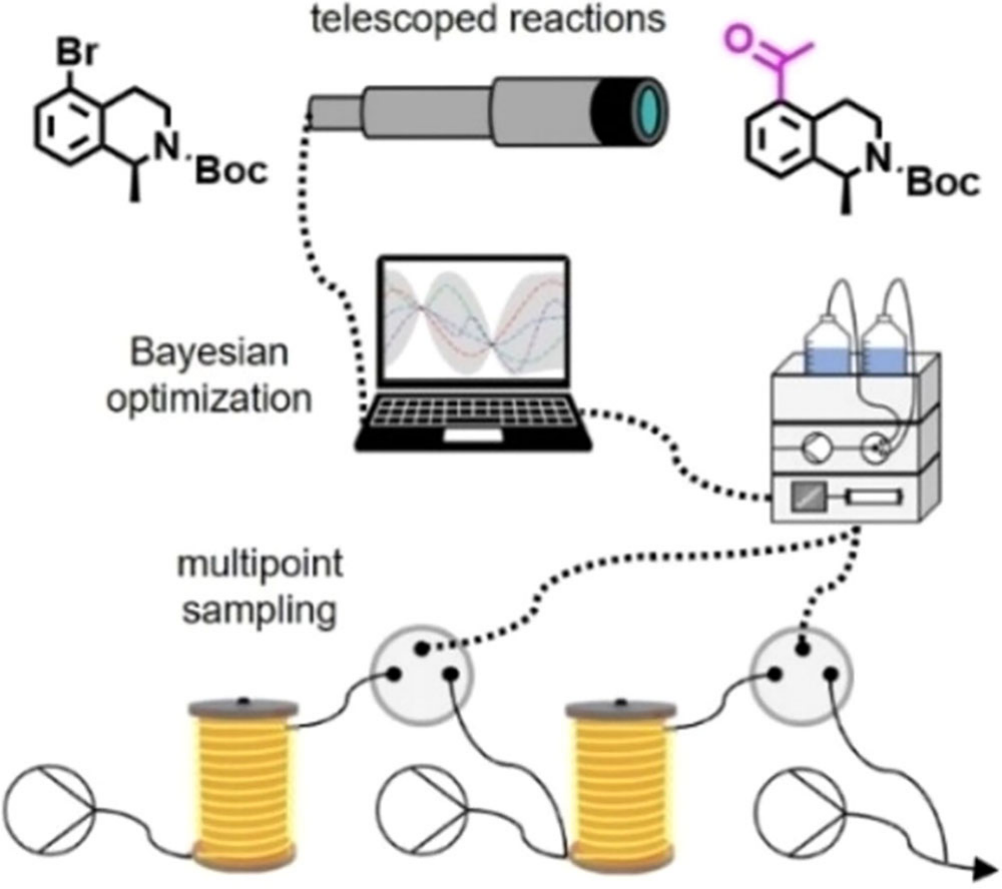
Autonomous continuous flow platform for the rapid development of multistep synthesis. "Reprinted from Clayton, A. D.; Pyzer-Knapp, E. O.; Purdie, M.; Jones, M. F.; Barthelme, A.; Pavey, J.; Kapur, N.; Chamberlain, T. W.; Blacker, A. J.; Bourne, R. A. Bayesian self-optimization for telescoped continuous flow synthesis. Angew. Chem. Int. Ed. 62 (3), e202214511, Copyright (2023), with permission from John Wiley & Sons. License CC BY 3.0 (https://creativecommons.org/licenses/by/3.0)”.

The application of reaction optimization has been limited in many cases to single-step reactions, multiobjective or mixed variable conditions. However, for complex molecules that require various coupling steps, the optimization of the reaction parameters for each coupling reaction becomes challenging. In telescoped reactions, the combination of individually optimized reaction conditions cannot be considered, as formation or consumption of key intermediates plays a critical role. All variables should be optimized simultaneously to relate the effect of each variable to the reaction outcome. In this regard, Bourne, Clayton and coworkers developed a telescoped continuous flow synthesis with Bayesian self-optimization for the preparation of arylketones (Fig. 9)^212^. A Bayesian optimization algorithm with an adaptive expected improvement acquisition function (BOAEI) was selected with nine Latin hypercube (LHC) experiments for 23 sequential iterations. The multistep reaction was based on a Heck coupling-intramolecular cyclization reaction and selective deprotection steps, which accounted for an optimal overall yield of 81% in continuous flow in just 13 experiments. The model identified the best reaction conditions for long residence times, high equivalents of the alcohol partner and moderate temperatures. Remarkably, it was able to point out the lower influence of *p*-toluenesulphonic acid (TsOH) in the reaction yield in comparison with the rest of the parameters. In addition, HPLC was installed for multipoint sampling to quantify reaction mixtures and impurities for reaction monitoring and understanding.

The possibility of integrating biological platforms to screen the activity of the molecules has been described some years ago for some flow homogeneous catalytic transformations^197^. Under this approach, not only can the synthesis and purification of the organic derivatives be accomplished, but also the measurement of biological parameters to build SAR data. The development of this equipment allows to decrease importantly the response time between the synthesis and the activity of the molecules to accelerate drug discovery processes. A recent contribution was described by Guo and coworkers for the rapid identification of bioactive molecules in a screening platform for protein-directed dynamic combinatorial chemistry^213^. Again, the Suzuki cross-coupling served to synthesize a library of compounds to further identify the best binders by MS analysis. A better interaction between the candidates and the protein was observed as a consequence of the improved mass transfer in continuous flow. This type of integrated flow system clearly reduces the time between the synthesis and analysis of the drug candidates to further explore more chemical escape and elaborate SAR data.

**Fig. 10:**
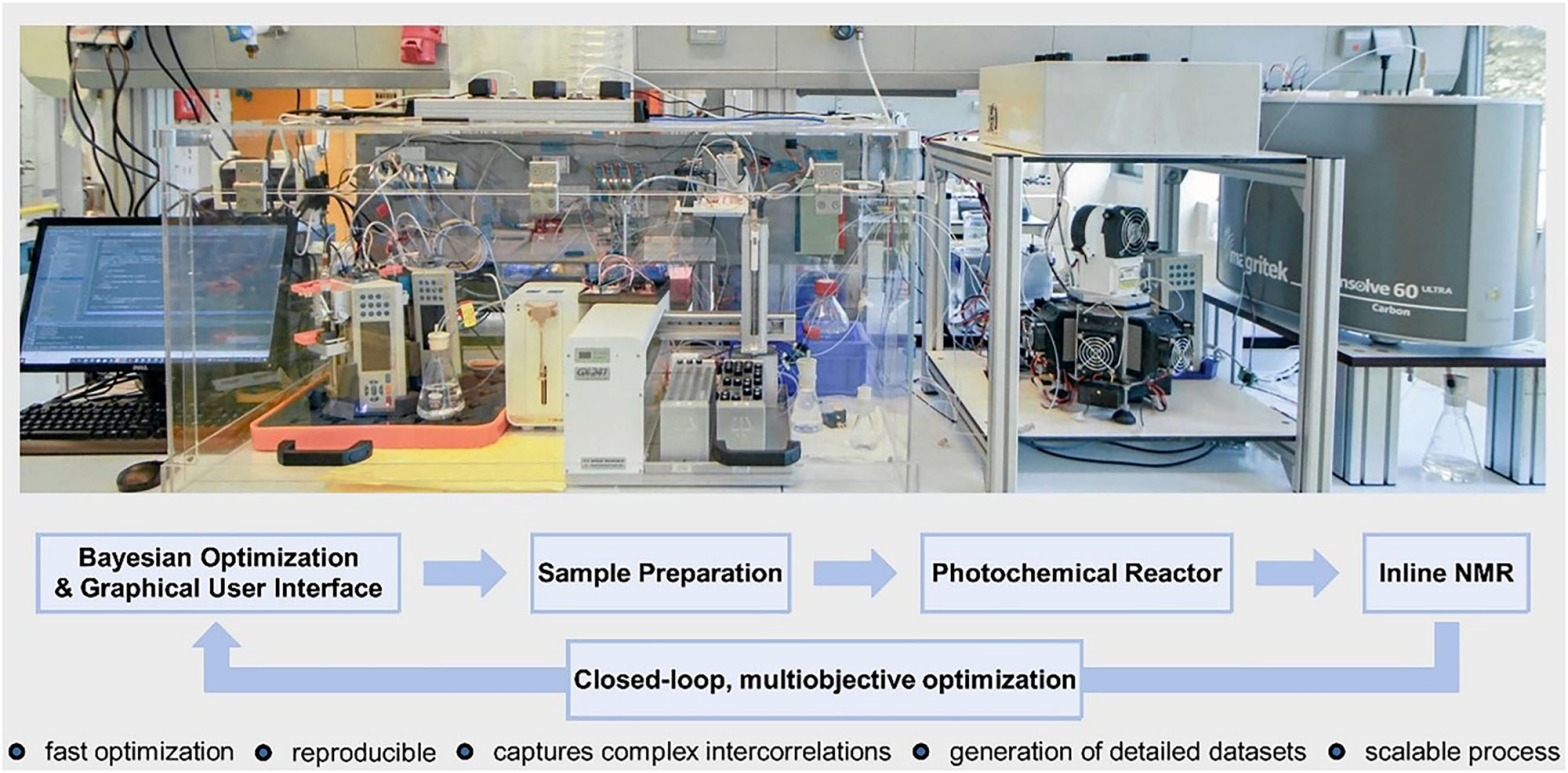
Photograph and workflow of RoboChem. "Reprinted from Slattery, A.; Wen, Z.; Tenblad, P.; Sanjose-Orduna, J.; Pintossi, D.; den Hartog, T.; Noel, T. Automated self-optimization, intensification, and scale-up of photocatalysis in flow. Science, 383, 1220–1225, Copyright (2024), with permission from American Association for the Advancement of Science. License 1648477-1”.

Beyond individual case studies, the combination of homogeneous catalysis, continuous flow and integrated technologies has enabled the development of automated and high-throughput platforms. Photochemistry offers notable benefits to rapidly expand the chemical space^81,214,215^. A particularly notable contribution comes from Noël and colleagues, who developed a platform based on photochemical microslug experiments (Fig. 10)^216^. This machine learning platform, utilizing Bayesian optimization, minimizes the number of experiments needed to achieve optimal conditions. The authors successfully applied this platform, known as Robochem, to a variety of reactions, including C–H photocatalytic HAT alkylation and trifluoromethylthiolation, oxytrifluoromethylation of alkenes via photocatalytic single-electron transfer, aryl trifluoromethylation, and C(sp^2^)–C(sp^3^) cross-electrophile coupling. They employed inline NMR as a process analytical tool, though they noted that other common inline analytical instruments could be substituted. Excellent manuscripts dedicated to process analytical technology also highlighted the specific needs of flow photochemical reactions^18,119^.

**Fig. 11:**
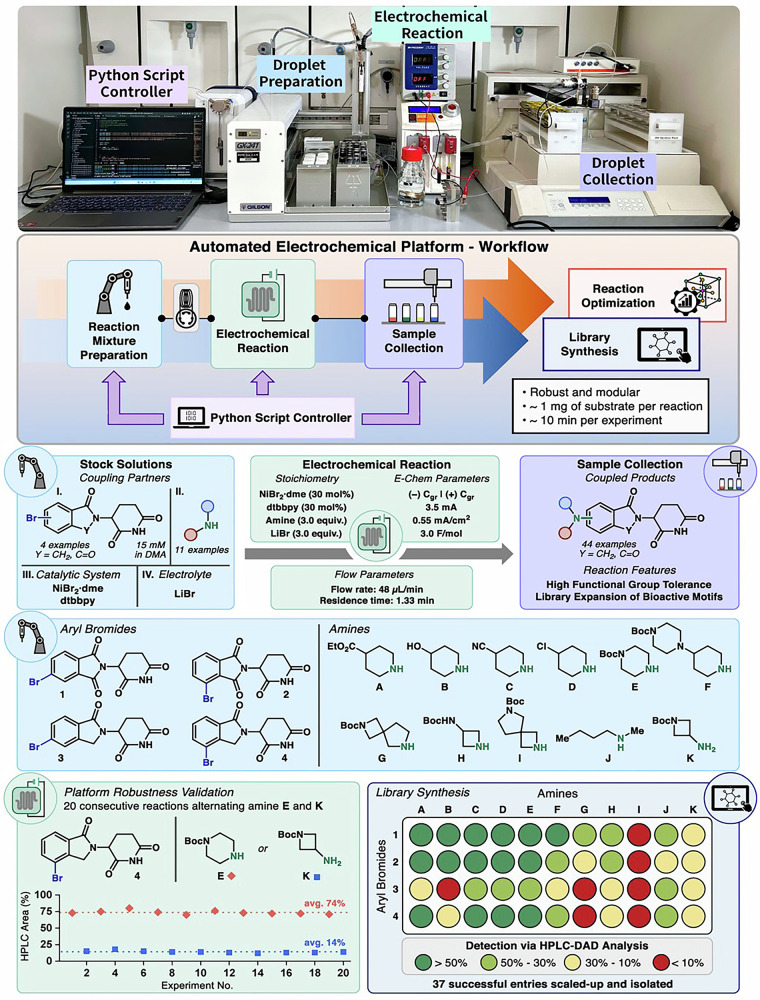
(Top) Picture of the setup and workflow schematization. (Center) Stock solutions, electrochemical reaction conditions, and reagents for the library synthesis. (Bottom) Platform robustness and library synthesis. "Reprinted from Rial-Rodríguez, E.; Williams, J. D.; Cantillo, D.; Fuchß, T.; Sommer, A.; Eggenweiler, H.-M.; Kappe, C. O.; Laudadio, G. An automated electrochemical flow platform to accelerate library synthesis and reaction optimization. Angew. Chem. Int. Ed., 63 (51), e202412045, Copyright (2024), with permission from Wiley-VCH GmbH”.

Similar automated approaches have been adapted in the field of electrocatalysis, which has been incorporated into automated systems and reaction optimization. Eggenweiler, Kappe and Laudadio reported an automated system able to carry out C–N bond formations through Buchwald-Hartwig and Ullman reactions catalyzed by Ni complexes through a gas-separated reaction slug approach to exclude dispersion, avoid cross-contaminations and minimize material consumption. Stock solutions were prepared, and the reactions took place in an operator-free manner to obtain reproducible data. The electrochemical reaction provided a set of data points that were analyzed by a software/hardware combination. This innovative automated platform was applied for reaction optimization and library synthesis via DoE to obtain a set of 44 compounds with minimal human intervention^195^. Even a challenging transformation with a low initial yield (6%) was rapidly optimized using DoE; within just 4 h and 23 experiments, product formation increased sixfold, demonstrating the high efficiency of the platform (Fig. 11).

Continuous efforts in this direction have achieved an autonomous platform that combines Bayesian optimization with real-time analytical monitoring. In a preprint, the same authors have reported a slug-based strategy showcasing the benefits of this closed-loop automation in electrochemistry through three distinct case studies: a nickel-catalyzed C–N cross-coupling, anodic functionalization of an amino acid, and decarboxylative alkylation of a natural product^217^. Each reaction was optimized by exploring a wide range of chemical parameters, encompassing both continuous and categorical variables. The optimized conditions were subsequently translated from slug flow to continuous flow to facilitate product isolation.

In summary, the integration of automation HTE with continuous flow chemistry is redefining the pace and scope of reaction development. Miniaturized nanomole screening^54,218^, coupled with reduced susceptibility to human error^200^, enables the rapid generation of high datasets that feed directly into machine learning driven optimization. Beyond efficiency, sustainable benefits such as lower carbon footprint, reduced chemical waste and decreased energy consumption are obtained through modular reactor designs^219^, which accelerate time-to-market for essential compounds^220,221^.

However, the high initial cost and complexity of these platforms are still a challenge^218,221^. A closer collaboration among various disciplines, including chemists, chemical engineers, and data scientists, is needed to overcome these limitations. While machine learning (ML) and artificial intelligence (AI) tools offer promising avenues for synthesis planning and process development^220,222^, their generalizability across different types of organic reactions remains a challenge ^223,224^ due to factors like data sparsity and the inconsistent quality of existing datasets. Future work will need to focus on developing hybrid models that combine data-driven approaches with expert-encoded rules^222^. For more widespread adoption, the development of user-friendly and open-access databases with standardized data representations is paramount to democratize access in automation^223,224^. The future success of automated chemistry hinges on continued collaboration between academia, industry, and equipment vendors, coupled with sustained investment in research and development to foster a cohesive ecosystem to drive innovation and overcome current limitations.

## Outlook

Homogeneous catalysis has been incorporated in continuous flow chemistry to overcome some limitations found in batch mode. This tendency has even been improved by integrating novel technologies such as photo- and electrochemistry, to transform the landscape of synthetic chemistry through new levels of selectivity, efficiency and discovery. Automated platforms have facilitated the development of homogeneous catalytic processes without human intervention, reducing costs and time while enhancing reproducibility. In some cases, these methodologies have integrated software or reaction optimization and library synthesis. Recent efforts are based on merging novel synthetic approaches with automated systems that could give rise to autonomous platforms led by decision-making algorithms. We expect an enormous growth during the following years in the field, as the potential of preparing organic molecules by integrating software and synthetic technologies in automated systems provides a wide range of diversity desirable for both synthetic and medicinal chemists.
